# Comparative Effects of Thymoquinone, Tranexamic Acid, and Porcine Dermal Collagen on Seroma Formation and Tissue Remodeling After Mastectomy in a Rat Model

**DOI:** 10.3390/medicina62071228

**Published:** 2026-06-24

**Authors:** Ali Duran, Nelin Hacioglu, Aylin Turkoglu Dulger, Feray Kockar, Esra Tokay, Eren Altun, Ferhat Cay, Azad Gazi Sahin, Huseyin Pulat, Murat Basbug

**Affiliations:** 1 Department of Surgery, Faculty of Medicine, Balıkesir University, Balikesir 10145, Turkey; 2 Department of Molecular Biology and Genetics, Faculty of Sciences and Arts, Balıkesir University, Balikesir 10145, Turkey; 3 Department of Pathology, Bagcılar Training and Research Hospital, University of Health Sciences, Istanbul 34200, Turkey; 4 Mersin City Hospital, General Surgery Clinic, Mersin 33000, Turkey

**Keywords:** seroma formation, mastectomy, axillary dissection, thymoquinone, tranexamic acid, porcine dermal collagen

## Abstract

*Background and Objectives*: Seroma formation is the most common postoperative complication following mastectomy and axillary dissection, negatively affecting wound healing and delaying adjuvant therapy. Despite numerous surgical and pharmacological approaches, no universally effective strategies have been established. This study aimed to comparatively evaluate the effects of porcine dermal collagen (PDC), tranexamic acid (TXA), and thymoquinone (TQ) on seroma formation and tissue repair. *Materials and Methods*: A randomized controlled experimental study was conducted using 40 female Wistar albino rats that underwent modified radical mastectomy and axillary dissection. All surgical and postoperative procedures were performed in accordance with the institutional animal welfare and ethical guidelines, including postoperative analgesic administration. The animals were divided into four groups: control, PDC, TXA, and TQ (*n* = 10 each). Seroma volume was measured on postoperative day 14. Histopathological evaluation, immunohistochemical analysis (FGF2, VEGF, TGF-β1, p53), and quantitative real-time PCR were performed to assess tissue remodeling and molecular responses. *Results*: All treatment groups demonstrated a significant reduction in seroma volume compared to the control group, with the most pronounced decrease observed in the TQ and TXA groups (*p* < 0.0001), while PDC showed a moderate effect (*p* < 0.01). Histopathological analysis revealed increased collagen deposition and fibrin formation in the PDC and TQ groups, whereas TXA exhibited a more limited remodeling profile than the others. Immunohistochemical and molecular analyses showed significant upregulation of VEGF across all groups, with broader and more consistent increases in the PDC and TQ groups. TGF-β1 and FGF2 expression demonstrated region-specific increases, particularly in the thoracic tissue. p53 expression remained relatively stable in the TXA group but was elevated in specific regions in the PDC and TQ groups. Importantly, the increased inflammatory infiltration, edema, vascular proliferation, and fibrin deposition observed in the TQ group may reflect not only active tissue remodeling processes but also prolonged inflammatory activation and enhanced fibrotic responses and should therefore be interpreted cautiously. *Conclusions*: PDC, TXA, and TQ differentially modulate postoperative seroma formation via distinct biological mechanisms. While TXA primarily exerts a targeted anti-seroma effect and PDC enhances extracellular matrix stabilization, TQ is associated with broader angiogenic, inflammatory, and tissue remodeling responses within this preclinical rat model. These findings should be considered exploratory and hypothesis-generating, and additional mechanistic studies and clinical investigations are necessary before definitive therapeutic conclusions can be established regarding the use of TQ in human breast surgery settings.

## 1. Introduction

Mastectomy and axillary dissection remain essential surgical procedures for breast cancer management, providing effective local disease control. However, despite advances in surgical techniques, postoperative complications continue to negatively affect patient recovery and their quality of life. Seroma formation is one of the most common early complications after mastectomy and is characterized by the accumulation of serous fluid in the axillary space or subcutaneous flaps [1,2]. The wide range of reported incidence rates suggests that seroma formation has a multifactorial pathophysiology that is not yet fully understood [2].

While seroma is generally benign, it can lead to significant clinical consequences, including the need for repeat aspiration, risk of infection, delayed wound healing, and delayed adjuvant therapy [2]. Studies on the biochemical and cellular properties of seroma fluid suggest that its formation mechanism cannot be explained solely by lymphatic leakage and that the inflammatory response and immune cell infiltration play a significant role [1]. Inflammatory processes triggered by surgical trauma, cytokine release, increased vascular permeability, and fibrinolytic activity have been suggested as the key biological components of seroma development [1].

Surgical and pharmacological approaches to reduce seroma formation have been extensively researched; however, to date, no method has been demonstrated to provide universal and consistent success [3]. Although modalities such as the modification of dissection techniques, use of energy devices, and closed absorbent drainage are commonly applied in clinical practice, their effectiveness remains controversial [3,4]. Therefore, novel interventions targeting the underlying biological mechanisms of seroma formation are needed.

Porcine dermal collagen (PDC) has attracted interest in experimental models because of its potential to reduce necrotic areas and enhance the adhesion of tissue planes. Studies in rodent mastectomy models have shown that porcine dermal collagen can reduce seroma volume and promote tissue organization [5]. Tranexamic acid (TXA), thanks to its antifibrinolytic action, is able to reduce postoperative drainage volume and hematoma formation; a reduction in seroma incidence has also been reported in selected clinical studies [6,7].

Thymoquinone (TQ), a compound derived from Nigella sativa, exhibits potent anti-inflammatory and antioxidant properties. It has been shown to suppress NF-κB and pro-inflammatory cytokine pathways and modulate the wound healing process [8]. Owing to these biological properties, thymoquinone has been proposed as a potential modulator of postoperative inflammatory and tissue remodeling responses following surgical injury.

To better understand the pathogenesis of seroma at the molecular level, it is necessary to assess not only macroscopic fluid volume but also the gene expression profiles associated with inflammation and tissue remodeling. RNA isolation and quantitative real-time PCR (qRT-PCR) analysis enable precise determination of expression levels of inflammatory mediators, angiogenic factors, and fibrosis-associated genes in scar tissue [9,10,11]. This approach allows for the elucidation of the biological effects of interventions, not only on clinical outcomes but also at the molecular level of the body. In the present study, VEGF and FGF2 were selected because of their established roles in angiogenesis, vascular permeability, fibroblast activation, and granulation tissue formation during wound healing. TGF-β1 was included because of its central involvement in extracellular matrix remodeling, collagen deposition, fibrosis, and regulation of inflammatory responses. In addition, p53 was evaluated because of its role in cellular stress signaling, apoptosis regulation, cell cycle control, and tissue remodeling-associated cellular turnover following surgical injury. The combined assessment of these markers was intended to provide a broader overview of the angiogenic, fibrotic, inflammatory, and cellular regulatory pathways associated with postoperative wound healing and seroma formation.

Despite the growing interest in these therapeutic strategies, direct comparative evidence integrating macroscopic outcomes with tissue-level and transcriptional responses remains scarce. Most available studies have predominantly focused on gross seroma reduction, with limited insight into the coordinated biological processes underlying postoperative fluid accumulation and tissue remodeling. To address this gap, the present study was designed to systematically compare the effects of porcine dermal collagen, tranexamic acid, and thymoquinone within a unified experimental framework using a standardized rat mastectomy and axillary dissection model. In addition to the conventional assessment of seroma volume, we integrated histopathological, immunohistochemical, and quantitative real-time PCR analyses to delineate the molecular and cellular mechanisms governing postoperative wound healing. By combining multi-level analyses, this study aims to provide a mechanistic understanding of how these agents differentially modulate inflammation, angiogenesis, and extracellular matrix remodeling, thereby offering a more comprehensive framework for optimizing therapeutic strategies for seroma prevention.

## 2. Materials and Methods

### 2.1. Study Design, Randomization, and Ethical Approval

This study was conducted as a randomized, controlled, experimental animal investigation adhering to the ARRIVE 2.0 guidelines and institutional regulations concerning the care and use of laboratory animals. Ethical approval was obtained from the Balıkesir University Animal Experiments Local Ethics Committee (Approval No: 2020-5/6, Date: 20 August 2020). Animals were allocated to the experimental groups using a computer-generated randomization sequence prior to surgery to minimize allocation bias. A priori sample size calculation was performed using G*Power 3.1 software. Based on preliminary data, an anticipated effect size (f), α = 0.05, and statistical power (1 − β) = 0.80 were employed to detect differences in seroma volume between the groups, which was the primary endpoint. Accordingly, a minimum of nine animals per group was required. Ten animals were included in each group to account for potential perioperative losses.

### 2.2. Experimental Groups

Forty adult female Wistar albino rats with an average weight of 320 g (range: 300–340 g) were obtained from the Balıkesir University Experimental Animal Center. The animals were housed in pairs under standard environmental conditions (22–24 °C temperature, 55–60% humidity, and a 12 h light/dark cycle) with ad libitum access to standard laboratory chow and water. Rats were randomly allocated to four independent experimental groups (*n* = 10 per group) prior to surgery to minimize allocation bias and maintain standardized experimental conditions.

Group A (Control): Mastectomy + axillary dissection + 1 mL sterile saline irrigation
Group B (PDC): Mastectomy + axillary dissection + local application of porcine dermal collagen
Group C (TXA): Mastectomy + axillary dissection + local administration of tranexamic acid
Group D (TQ): Mastectomy + axillary dissection + local administration of thymoquinone (4 mg/kg in 1 mL solution)

In addition to postoperative tissue collection performed on postoperative day 14, limited baseline tissue samples (C0) were obtained intraoperatively from the same animals immediately after mastectomy and before treatment. These baseline samples were used solely as reference materials for comparative histopathological and molecular analyses and did not represent separate experimental groups. Therefore, all postoperative comparisons were performed within the same standardized experimental timeline, and the terms C0, C14, and T14 indicate analytical sampling time points rather than independent intervention groups (Supplementary Figure S9).

### 2.3. Surgical Procedure

Prior to surgery, the animals underwent a 12-hour fasting period. General anesthesia was induced using intraperitoneal ketamine (80 mg/kg) and xylazine (10 mg/kg). Adequate anesthesia depth was confirmed by the absence of a pedal withdrawal reflex. Postoperative analgesia was provided using paracetamol to minimize postoperative pain and distress associated with the surgical procedures. The animals were monitored daily throughout the postoperative period for general health status, wound healing, and humane welfare endpoints. Surgical procedures were performed under sterile conditions [9]. An incision was made along the midline, stretching from the jugular fossa to the xiphoid. The pectoralis major muscle was carefully removed, and axillary lymph node dissection was performed while preserving the major blood vessels. Hemostasis was achieved without lymphatic vessel ligation to allow consistent seroma formation across the groups. Baseline tissue samples were collected prior to treatment for molecular and histological analyses. Tranexamic acid (TXA) was obtained from Transamin^®^ (Bilim Pharmaceuticals, Istanbul, Türkiye), and thymoquinone (TQ) was purchased from Sigma-Aldrich (St. Louis, MO, USA), and porcine dermal collagen (PDC) was obtained from Pelnac^®^ (Gunze Limited, Kyoto, Japan). Following the application of group-specific treatments, the skin was closed using 4/0 polypropylene sutures (Prolene^®^, Ethicon, Johnson & Johnson, Raritan, NJ, USA).

### 2.4. Postoperative Evaluation

On postoperative day 14, the animals were re-anesthetized using the same protocol. Seroma fluid was aspirated under sterile conditions using calibrated syringes and recorded in milliliters. The animals were subsequently euthanized under deep anesthesia by cervical dislocation in accordance with institutional ethical guidelines [9]. Tissue samples were collected from the incision site, thoracic wall, and axillary region. Tissue sampling was anatomically standardized in all animals. Axillary samples (~0.5 cm^2^) were obtained from the central axillary fossa at the level of the third rib, skin samples (~0.5 cm^2^) were excised from the midline incision site, and thoracic wall samples (~0.5 cm^2^) were collected from the anterior thoracic wall at the level of the fourth intercostal space.

Separate tissue samples were collected for histopathological/immunohistochemical and molecular analyses to avoid cross-processing artifacts. Samples designated for histopathological and immunohistochemical evaluation were immediately fixed in 10% neutral buffered formalin, whereas samples designated for molecular analysis were immediately preserved in RNA stabilization solution and stored at −80 °C until further processing.

### 2.5. Histopathological and Immunohistochemical Analysis

Tissue specimens were fixed in 10% neutral buffered formalin for 24 h immediately after collection, routinely processed through a graded ethanol series and xylene, and embedded in paraffin. Sections (5 μm) were cut using a rotary microtome (Leica RM2235, Leica Biosystems, Nussloch, Germany). Paraffin-embedded tissues were sectioned into slices 5 μm thick and subsequently stained with hematoxylin and eosin (H&E) for histopathological examination. Additional sections were stained with Masson’s trichrome (Sigma-Aldrich, St. Louis, MO, USA) according to the manufacturer’s protocol to evaluate collagen deposition. The parameters assessed included inflammatory cell infiltration, fibroblast proliferation, collagen deposition, vascular congestion/proliferation, fibrin formation and necrosis. Scoring was performed using a modified Ehrlich–Hunt scale ranging from 0 to 4. The scoring criteria were as follows: 0 = absent; 1 = mild (focal, <25% of field); 2 = moderate (multifocal, 25–50%); 3 = marked (diffuse, 50–75%); 4 = severe (>75%). The “vascular congestion/proliferation” parameter was scored as a composite because passive engorgement and active capillary sprouting co-occur during early wound repair and cannot be reliably separated using H&E staining alone. For each specimen, a minimum of five high-power fields (HPFs) were randomly selected for evaluation, and mean scores were calculated. A pathologist blinded to the group assignments conducted the evaluation. Images were acquired using a light microscope (BX53; Olympus Corporation, Tokyo, Japan) fitted with a digital camera (DP74; Olympus) and CellSens Standard version 2.3 (Olympus Corporation, Tokyo, Japan). Two sections per animal were evaluated, five HPFs per section were scored, and the mean score per animal was used for the statistical analysis. All animals in each group were analyzed individually. Intraobserver reproducibility was confirmed by re-scoring 20% of randomly selected slides after a two-week interval, and intraclass correlation coefficients exceeded 0.85 for all parameters. Immunohistochemical staining for FGF2, VEGF, TGF-β1, and p53 was executed using the avidin–biotin–peroxidase complex method [9,10,11]. Following deparaffinization and rehydration, heat-induced antigen retrieval was performed using citrate buffer (pH 6.0) at 95 °C for 20 min. Endogenous peroxidase activity was blocked with 3% hydrogen peroxide for 10 min. Sections were then incubated overnight at 4 °C with the following primary antibodies diluted in Dako antibody diluent: anti-FGF2 (ab18408, Abcam, Cambridge, UK; 1:200), anti-VEGF (ab32152, Abcam; 1:200), anti-TGF-β1 (ab215715, Abcam; 1:150), and anti-p53 (ab26, Abcam; 1:100). Detection was performed using a biotinylated secondary antibody and streptavidin-HRP detection system (LSAB+ System-HRP, Dako/Agilent, Santa Clara, CA, USA), followed by visualization with 3,3′-diaminobenzidine (DAB) chromogen and hematoxylin counterstaining. The H-score was determined by multiplying the percentage of positively stained cells by the staining intensity (0–4), resulting in a total score between 0 and 300.

### 2.6. Molecular Analysis

Total RNA was isolated using TRIzol™ Reagent (Invitrogen, Carlsbad, CA, USA) following the manufacturer’s guidelines. The RNA concentration and purity were assessed using spectrophotometry. A reverse transcription kit was used to synthesize cDNA from 1 μg of total RNA, following the manufacturer’s instructions. Primers for FGF2, VEGF, TGF-β1, and p53 were designed using the NCBI Primer-BLAST tool. The primer sequences were as follows: TGF-β1: 5′GCTCAG TCT GTC TAC CTG CA-3′, R: 5′-GGC GGG ATG GCA TCA AGG TA-3′; FGF2, F: 5′-ACT TCG CTT CCC GCA CTG C-3′, R: 5′-CCA GTT GGT ATG TGG CAC TG-3′, VEGF F: 5′-CCCATGAAGT GGTGAAGTTC-3′, R: 5′-GAACAAGGCTCACAGTGAAC-3′; p-53, F: 5′-AGA CAT TTT CAT GCT TAT GG-3′, R: 5′-ACC ATC AGA GCA ACG CTC AT-3′; and GAPDH, F: 5′CTG GAG AAA CCT GCC AAG TAT G-3′, R: 5′-GGT GGA AGA ATG GGA GTT GCT-3′.

qRT-PCR was conducted with SYBR Green in a total reaction volume of 10 μL, which included 5 μL of SYBR Green Master Mix, 1 μL of cDNA, 0.5 μL of forward primer, 0.5 μL of reverse primer, and 3 μL of nuclease-free water. The thermal cycling conditions consisted of an initial denaturation step at 95 °C for 10 min, followed by 40 amplification cycles of denaturation at 95 °C for 15 s and annealing/extension at 60 °C for 60 s. Melting curve analysis was performed at the end of each run to confirm the amplification specificity. All reactions were performed in technical triplicates, with each experimental group comprising independent biological replicates (*n* = 10). Relative mRNA expression levels were normalized to GAPDH, serving as the endogenous control gene, and calculated using the 2^−ΔΔCt^ method (Livak and Schmittgen). Results were presented as fold changes relative to the control group [12,13,14,15].

### 2.7. Statistical Analysis

All statistical analyses were performed using GraphPad Prism 9.0. Data normality was checked using the Shapiro–Wilk test before selecting the appropriate comparative tests. Normally distributed variables were compared using one-way analysis of variance (ANOVA) with Tukey’s post hoc test, whereas non-normally distributed variables were analyzed using the Kruskal–Wallis test with Dunn’s correction. All statistical tests were two-tailed, and *p* < 0.05 was considered statistically significant.

## 3. Results

### 3.1. Histopathological and Immunohistochemical Findings

Histopathological and immunohistochemical analyses were performed to evaluate tissue-level changes following porcine dermal collagen (PDC), tranexamic acid (TXA), and thymoquinone (TQ) treatment in the axillary, skin, and thoracic regions (Supplementary Figures S1–S8). Semi-quantitative scoring of inflammatory and remodeling-related parameters, along with immunohistochemical assessment of FGF2, VEGF, TGF-β1, and p53 expression, was performed in all experimental groups. The results were presented comparatively according to the treatment groups and anatomical regions of interest.

**Figure 1:**
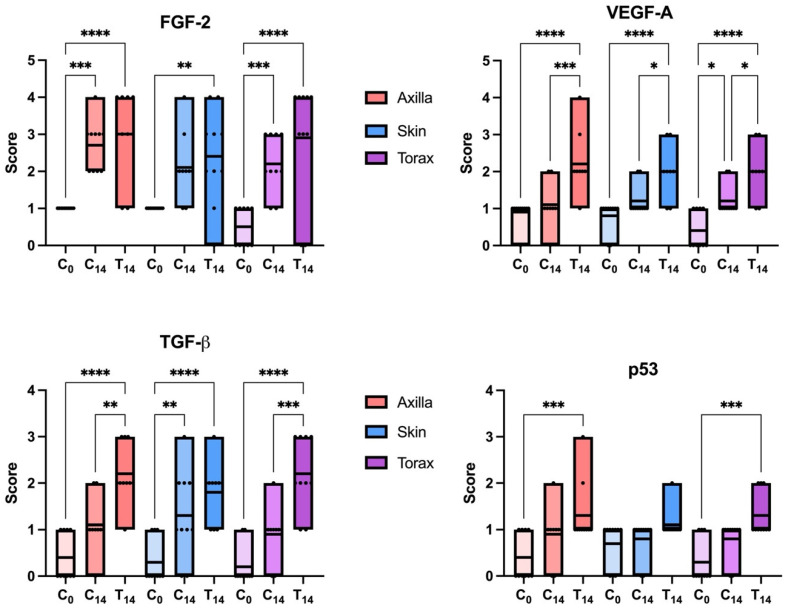
Histopathological and immunohistochemical evaluation of growth factor expression in the porcine dermal collagen (PDC) group. Semi-quantitative scoring of FGF-2, VEGF-A, TGF-β1, and p53 expression in axillary, skin, and thoracic tissues following modified radical mastectomy and axillary dissection. The immunohistochemical expression levels in porcine dermal collagen (PDC)-treated tissues were compared with those in the corresponding control tissues within each anatomical region. Data are presented as mean ± standard deviation (SD) (*n* = 10 animals per group). Statistical analyses were performed using one-way analysis of variance (ANOVA) followed by Tukey’s post hoc test. * *p* < 0.05, ** *p* < 0.01, *** *p* < 0.001 and **** *p* < 0.0001 versus the corresponding control group.

Histopathological and immunohistochemical analyses revealed region-specific changes in growth factor expression following porcine dermal collagen (PDC) treatment (Figure 1). FGF2 expression showed a moderate increase on postoperative day 14 in both the control (C_14_) and treated (T_14_) groups compared to baseline (C_0_) across all anatomical regions. However, no statistically significant differences were observed between C_14_ and T_14_ within the axillary, skin, or thoracic tissues. VEGF-A expression was significantly elevated at day 14 in both control and treated groups across all anatomical regions compared to baseline (*p* < 0.05–0.0001). Notably, VEGF-A levels in the treated group (T_14_) remained significantly higher than the baseline and showed a trend toward further increase compared to C_14_, particularly in the axillary and thoracic tissues. TGF-β expression demonstrated a significant increase at day 14 in both control and treated groups across all regions (*p* < 0.01–0.0001). The treated group (T_14_) exhibited higher expression levels than C_14_, especially in the thoracic tissue, although the magnitude of increase varied between regions. p53 expression showed a significant increase at day 14 compared to baseline in both the control and treated groups (*p* < 0.001), with a more pronounced elevation observed in the treated group (T_14_) in the axillary and thoracic tissues. No statistically significant difference was observed between C_14_ and T_14_ in the skin regions.

**Figure 2:**
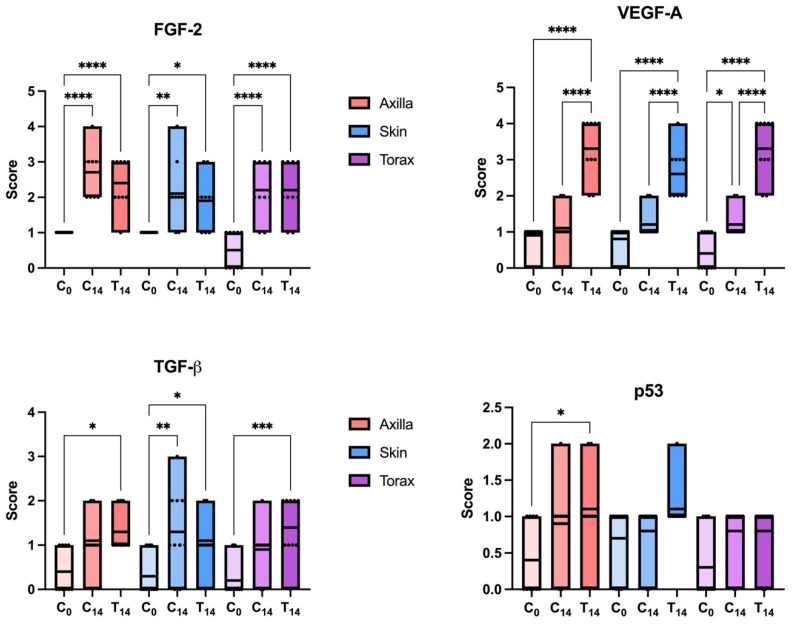
Histopathological and immunohistochemical evaluation of growth factor expression in the tranexamic acid (TXA) group. Semi-quantitative scoring of FGF-2, VEGF-A, TGF-β1, and p53 expression in axillary, skin, and thoracic tissues following modified radical mastectomy and axillary dissection. The immunohistochemical expression levels in the tranexamic acid (TXA)-treated tissues were compared with those in the corresponding control tissues within each anatomical region. Data are presented as mean ± standard deviation (SD) (*n* = 10 animals per group). Statistical analyses were performed using one-way analysis of variance (ANOVA) followed by Tukey’s post hoc test. * *p* < 0.05, ** *p* < 0.01, *** *p* < 0.001 and **** *p* < 0.0001 versus the corresponding control group.

Histopathological and immunohistochemical analyses revealed region-specific alterations in growth factor expression following tranexamic acid (TXA) treatment (Figure 2). FGF2 expression showed no statistically significant difference between the baseline (C_0_), postoperative day 14 control (C_14_), and treated (T_14_) groups in the axillary and thoracic tissues. In the skin region, FGF2 expression was significantly increased on day 14 compared to baseline (*p* < 0.05–0.01), with similar levels observed between C_14_ and T_14_. VEGF-A expression was significantly elevated at day 14 in both control and treated groups across all anatomical regions compared to baseline (*p* < 0.05–0.0001). The increase was particularly pronounced in the axillary and thoracic tissues, whereas a moderate elevation was observed in the skin region. TGF-β expression demonstrated a significant increase at day 14 compared to baseline in all regions (*p* < 0.05–0.001). The magnitude of increase was higher in the skin and thoracic tissues, whereas the axillary tissue showed a relatively moderate elevation. p53 expression showed no statistically significant differences between the C_14_ and T_14_ groups in any anatomical region. A modest increase was observed on day 14 compared to baseline, particularly in the axillary region (*p* < 0.05), whereas the skin and thoracic tissues remained relatively stable.

**Figure 3:**
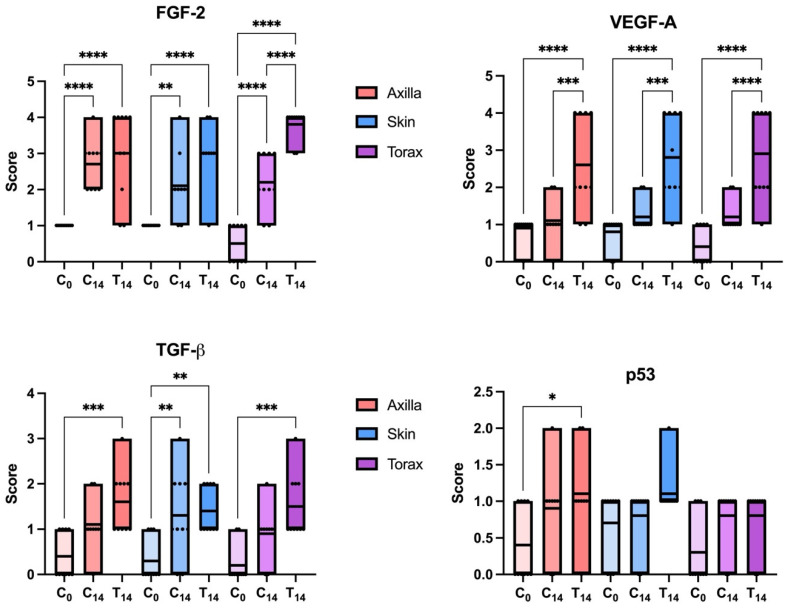
Histopathological and immunohistochemical evaluation of growth factor expression in the thymoquinone (TQ) group. Semi-quantitative scoring of FGF-2, VEGF-A, TGF-β1, and p53 expression in axillary, skin, and thoracic tissues following modified radical mastectomy and axillary dissection. The immunohistochemical expression levels in the thymoquinone (TQ)-treated tissues were compared with those in the corresponding control tissues within each anatomical region. Data are presented as mean ± standard deviation (SD) (*n* = 10 animals per group). Statistical analyses were performed using one-way analysis of variance (ANOVA) followed by Tukey’s post hoc test. * *p* < 0.05, ** *p* < 0.01, *** *p* < 0.001 and **** *p* < 0.0001 versus the corresponding control group.

Histopathological and immunohistochemical analyses revealed pronounced region-specific alterations in growth factor expression following thymoquinone (TQ) treatment (Figure 3). FGF2 expression showed a significant increase at postoperative day 14 in all anatomical regions compared to baseline (C_0_) (*p* < 0.01–0.0001). The increase was most prominent in the thoracic tissue, while the axillary and skin regions also demonstrated marked elevation at both control (C_14_) and treated (T_14_) time points. VEGF-A expression was significantly elevated at day 14 in all regions in both control and treated groups compared to baseline (*p* < 0.001–0.0001). The highest expression levels were observed in the treated group (T_14_), particularly in the thoracic and skin tissues. TGF-β expression demonstrated a significant increase at day 14 compared to baseline across all anatomical regions (*p* < 0.01–0.001). The increase was more pronounced in the skin and thoracic tissues, with both the C_14_ and T_14_ groups showing elevated expression levels. p53 expression showed a modest but significant increase at day 14 compared to baseline in the axillary region (*p* < 0.05), whereas the skin and thoracic tissues did not demonstrate statistically significant changes between time points.

**Figure 4:**
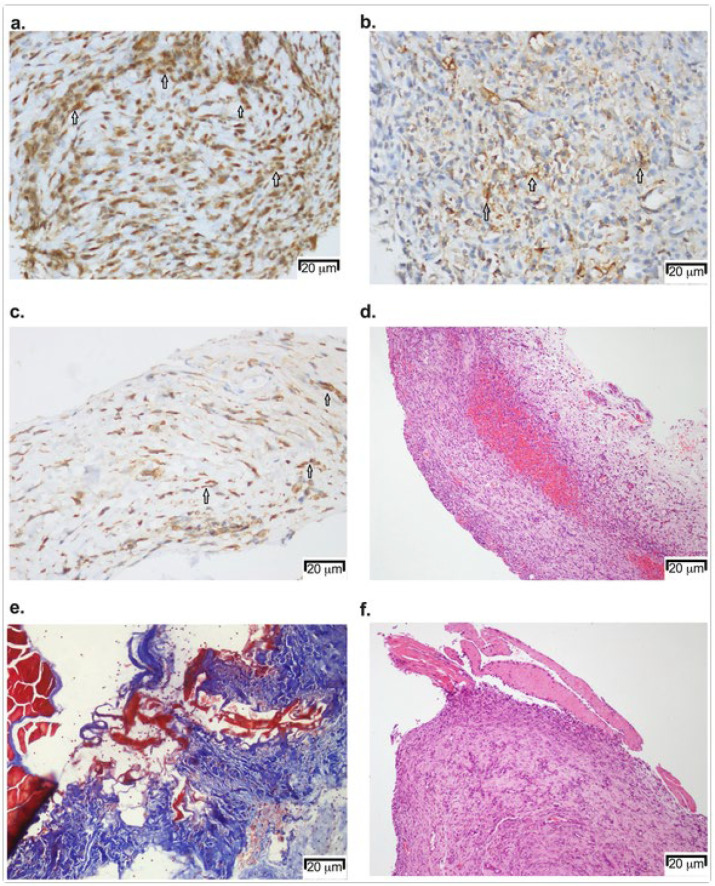
Representative immunohistochemical and histopathological images of axillary, skin, and thoracic tissues following modified radical mastectomy and axillary dissection. (**a**) Immunohistochemical staining for VEGF in thoracic tissue of the thymoquinone (TQ) group, demonstrating strong cytoplasmic positivity in vascular and stromal cells (arrows) (400×, anti-VEGF). (**b**) Immunohistochemical staining for TGF-β1 in thoracic tissue of the porcine dermal collagen (PDC) and TQ groups, showing increased stromal and extracellular positivity associated with collagen remodeling (arrows) (400×, anti-TGF-β1). (**c**) Immunohistochemical staining for FGF2 in thoracic tissue of the TQ group, demonstrating increased positivity in fibroblast-rich granulation tissue areas (arrows) (400×, anti-FGF2). (**d**) Hematoxylin and eosin (H&E) staining of axillary tissue in the TQ group, showing dense inflammatory cell infiltration, vascular congestion, and focal hemorrhagic regions (100× magnification). (**e**) Masson’s trichrome staining of thoracic tissue in the PDC and TQ groups, illustrating increased collagen deposition and extracellular matrix organization (200×). (**f**) Hematoxylin and eosin (H&E) staining of skin and thoracic tissues in the PDC and TQ groups, demonstrating fibrin accumulation, fibroblast-rich granulation tissue formation, and disruption of normal tissue architecture (100×). The images shown are representative photomicrographs illustrating key qualitative findings. Complete quantitative histopathological and immunohistochemical data for all groups, regions, and markers are presented in Table 1 and Figure 1, Figure 2, Figure 3 and Figure 5.

Comparative histopathological analysis revealed distinct region-specific morphological alterations in the treatment groups (Figure 4). In the axillary region, inflammatory cell infiltration was significantly increased in all treatment groups compared to that in the control group, with the highest scores observed in the TQ group. Microscopically, inflammatory infiltrates consisted predominantly of dense mononuclear cell accumulation surrounding areas of fibrin deposition and vascular proliferation. In the PDC and TQ groups, fibroblast proliferation and early granulation tissue organization were more prominent, particularly in the thoracic tissue sections, which demonstrated increased collagen accumulation and fibrin organization. The TQ group additionally demonstrated marked edema, increased vascular congestion/proliferation, and focal hemorrhagic areas within both the skin and thoracic tissues, suggesting active but complex tissue remodeling responses. Newly formed capillary structures and expanded vascular spaces were more evident in the TQ-treated tissues than in the TXA group. In contrast, TXA-treated tissues generally exhibited comparatively limited extracellular matrix remodeling and lower collagen accumulation, despite reduced seroma volume. Fibrin deposition was especially prominent in the PDC and TQ groups, where fibrillar eosinophilic extracellular material was observed within the surgical dead space and adjacent connective tissue compartments. Although overt widespread necrosis was not detected, focal degenerative tissue changes and localized disruption of the normal tissue architecture were occasionally observed in regions with intense inflammatory infiltration and fibrin accumulation.

### 3.2. mRNA Expression Analysis

**Figure 5:**
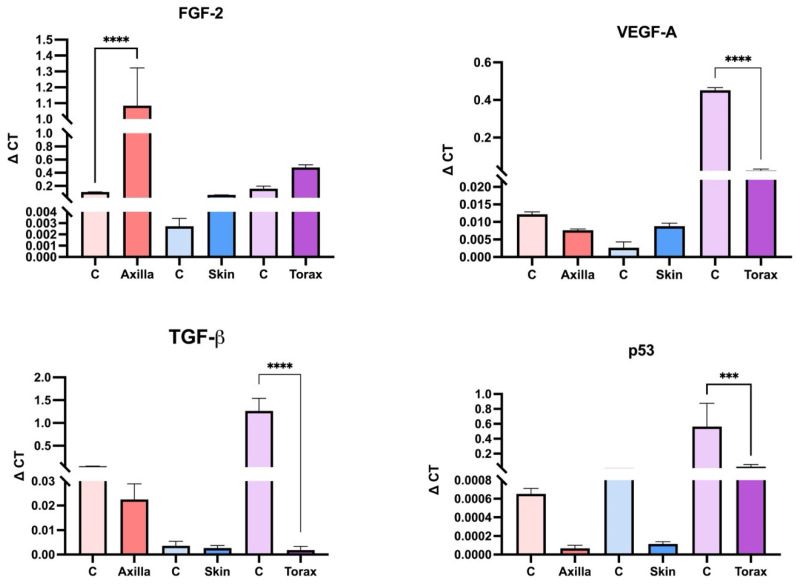
Relative mRNA expression levels of FGF-2, VEGF-A, TGF-β1, and p53 in porcine dermal collagen (PDC)-treated tissue. Quantitative real-time PCR analysis of FGF-2, VEGF-A, TGF-β1, and p53 mRNA expression in axillary, skin, and thoracic tissues following modified radical mastectomy and axillary dissection. Relative gene expression levels in the porcine dermal collagen (PDC)-treated tissues were compared with those in the corresponding control tissues within each anatomical region. Gene expression levels were normalized to GAPDH and calculated using the 2^−ΔΔCt^ method. Data are presented as mean ± standard deviation (SD) (*n* = 10 animals per group). Statistical analyses were performed using one-way analysis of variance (ANOVA) followed by Tukey’s post hoc test. *** *p* < 0.001; **** *p* < 0.0001. versus the corresponding control group.

Relative mRNA expression analysis revealed region-specific alterations in gene expression following treatment with porcine dermal collagen (PDC) (Figure 5). FGF2 expression was significantly increased in the axillary tissue compared to that in the control group (*p* < 0.0001), whereas only minor changes were observed in the skin and thoracic tissues of the control group. VEGF expression was elevated in thoracic tissue, reaching statistical significance (*p* < 0.0001), whereas no significant differences were observed in the axillary and skin regions. TGF-β1 expression was markedly increased in thoracic tissues (*p* < 0.0001), with minimal variation in axillary and skin tissues. Similarly, p53 expression was significantly increased in the thoracic tissue (*p* < 0.001), whereas no statistically significant differences were detected in the axillary and skin regions of the tissue.

**Figure 6:**
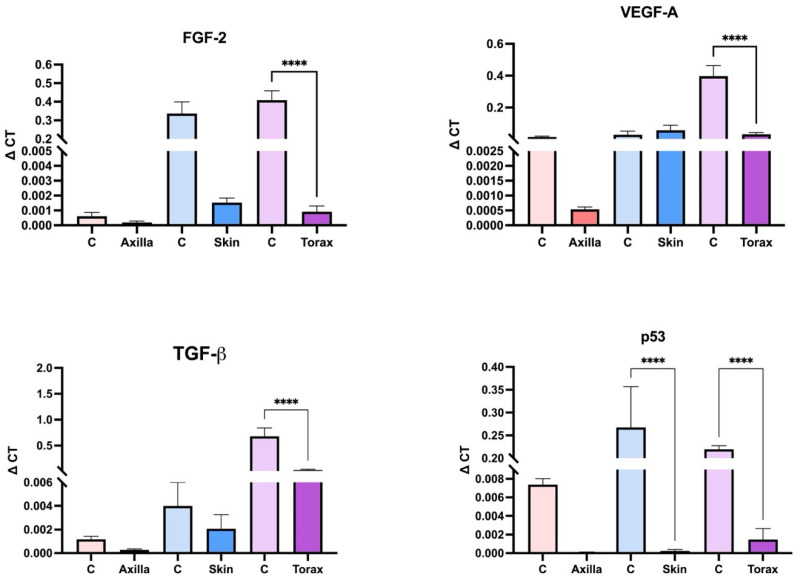
Relative mRNA expression levels of FGF-2, VEGF-A, TGF-β1, and p53 in thymoquinone (TQ)-treated tissue. Quantitative real-time PCR analysis of FGF-2, VEGF-A, TGF-β1, and p53 mRNA expression in axillary, skin, and thoracic tissues following modified radical mastectomy and axillary dissection. Relative gene expression levels in the thymoquinone (TQ)-treated tissues were compared with those in the corresponding control tissues within each anatomical region. Gene expression levels were normalized to GAPDH and calculated using the 2^−ΔΔCt^ method. Data are presented as mean ± standard deviation (SD) (*n* = 10 animals per group). Statistical analyses were performed using one-way analysis of variance (ANOVA) followed by Tukey’s post hoc test. **** *p* < 0.0001 versus the corresponding control group.

Relative mRNA expression analysis revealed region-specific alterations following thymoquinone (TQ) treatment (Figure 6). FGF2 expression was significantly increased in the axillary tissue compared to that in the control group (*p* < 0.0001), whereas no statistically significant differences were observed in the skin and thoracic tissues. VEGF expression was significantly elevated in thoracic tissue (*p* < 0.0001), whereas no significant differences were detected in axillary and skin regions. TGF-β1 expression was markedly increased in thoracic tissue (*p* < 0.0001), with no statistically significant changes observed in the axillary and skin tissues. Similarly, p53 expression was significantly increased in the thoracic tissue (*p* < 0.001), whereas no significant differences were observed in the axillary and skin regions of the body.

**Figure 7:**
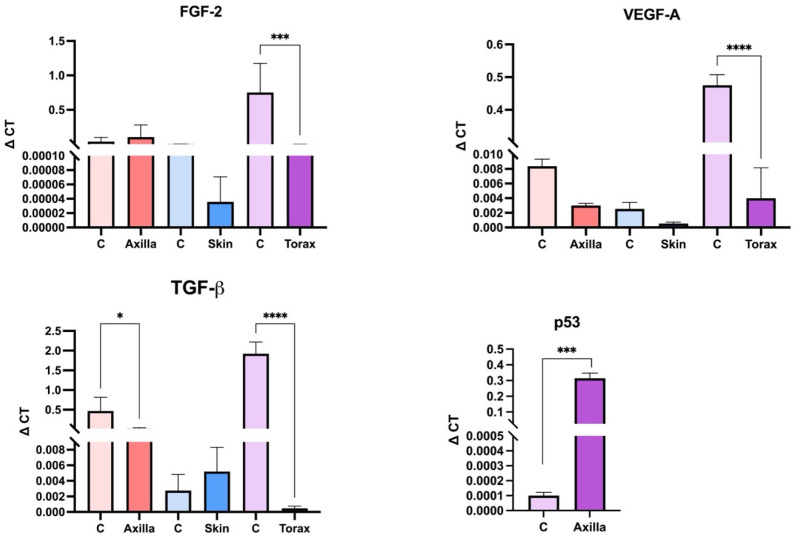
Relative mRNA expression levels of FGF-2, VEGF-A, TGF-β1, and p53 in tranexamic acid (TXA)-treated tissues. Quantitative real-time PCR analysis of FGF-2, VEGF-A, TGF-β1, and p53 mRNA expression in axillary, skin, and thoracic tissues following modified radical mastectomy and axillary dissection. Relative gene expression levels in tranexamic acid (TXA)-treated tissues were compared with those in the corresponding control tissues within each anatomical region. Gene expression levels were normalized to GAPDH and calculated using the 2^−ΔΔCt^ method. Data are presented as mean ± standard deviation (SD) (*n* = 10 animals per group). Statistical analysis was performed using one-way analysis of variance (ANOVA), followed by Tukey’s post hoc test. * *p* < 0.05, *** *p* < 0.001, and **** *p* < 0.0001 versus the corresponding control group.

Relative mRNA expression analysis demonstrated region-specific alterations following tranexamic acid (TXA) treatment (Figure 7). FGF2 expression was significantly increased in thoracic tissue compared to that in the control group (*p* < 0.001), whereas no statistically significant differences were observed in the axillary and skin tissues. VEGF expression was significantly elevated in the thoracic tissue (*p* < 0.0001), whereas no significant differences were detected in the axillary and skin regions. TGF-β1 expression was significantly increased in axillary and thoracic tissues (*p* < 0.0001), whereas no statistically significant difference was observed in the skin tissue. p53 expression was significantly increased in the axillary tissue (*p* < 0.001), whereas no significant differences were observed in other regions. Comparative analysis of relative mRNA expression across the treatment groups revealed distinct region-specific expression patterns for all examined genes (Figure 5, Figure 6 and Figure 7). FGF2 expression was significantly increased in the axillary tissue of the PDC group, whereas TXA and TQ treatments showed a significant elevation primarily in the thoracic tissue. VEGF expression was significantly elevated in the thoracic tissue in all treatment groups, with additional increases observed across multiple regions in the PDC and TQ groups, whereas TXA-induced changes were more restricted. TGF-β1 expression demonstrated a consistent and marked increase in thoracic tissue across all treatments, with additional elevation in the axillary tissue in the TXA group and broader regional increases in the PDC group. p53 expression was significantly upregulated in the thoracic tissue in the PDC group and in the axillary tissue in the TXA group, whereas TQ treatment resulted in increased expression in both skin and thoracic tissues. Overall, the distribution of gene expression changes varied across the treatment groups, with PDC and TQ demonstrating broader regional modulation, whereas TXA exhibited more region-specific effects than PDC and TQ.

### 3.3. Seroma Volume Findings

**Figure 8:**
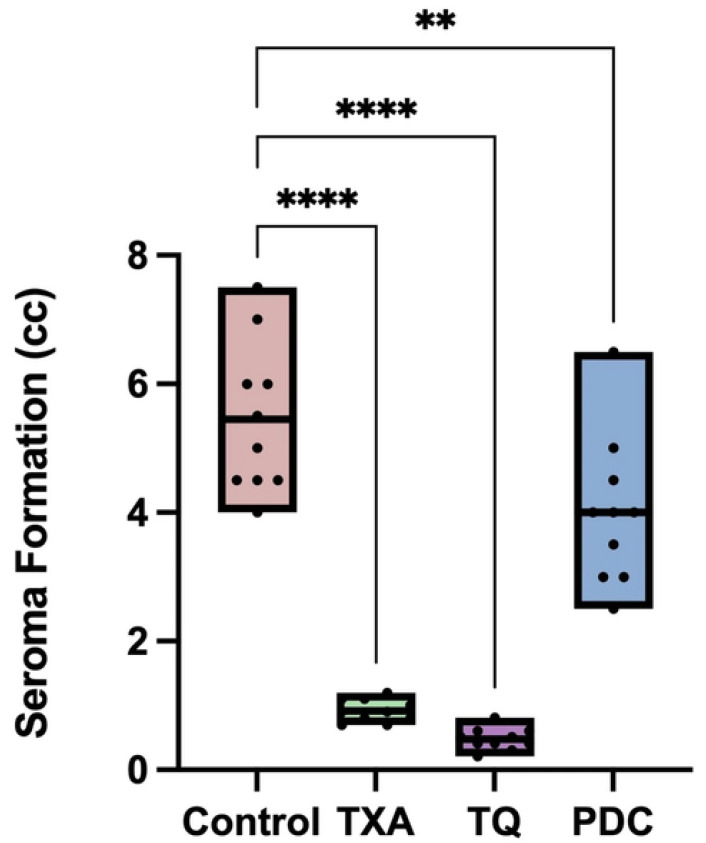
Comparison of seroma volumes following modified radical mastectomy and axillary dissection. Seroma volumes measured on postoperative day 14 in the control, tranexamic acid (TXA), thymoquinone (TQ), and porcine dermal collagen (PDC) groups are presented as box plots with individual data points (*n* = 10 per group). Box plots represent the median values and interquartile ranges. Statistical analysis was performed using one-way analysis of variance (ANOVA) followed by Tukey’s post hoc test. ** *p* < 0.01 and **** *p* < 0.0001 versus the control group. Detailed absolute seroma volume measurements, including mean ± SD, median (IQR), minimum-maximum values, and corresponding statistical comparisons with the control group, are provided in Supplementary Table S1.

Seroma volume analysis demonstrated significant differences among experimental groups (Figure 8). Compared with the control group, seroma volumes were significantly reduced in the TXA (*p* < 0.0001), TQ (*p* < 0.0001), and PDC (*p* < 0.01) groups. The lowest seroma volumes were observed in the TQ and TXA groups, whereas the PDC group showed a moderate reduction compared to the control group.

## 4. Discussion

Breast cancer is the most commonly diagnosed malignancy in women and a leading cause of cancer-related mortality worldwide. Even when evaluated irrespective of gender, it constitutes a substantial portion of the global cancer burden, ranking as the second most frequently diagnosed cancer and the fourth leading cause of cancer-related deaths in 2022 [16]. Surgical intervention, including modified radical mastectomy and axillary dissection, is the cornerstone of treatment for malignant and selected benign conditions. However, seroma formation remains the most common postoperative complication of breast surgery. Therefore, understanding the biological mechanisms underlying seroma formation and the effects of different therapeutic strategies is essential for improving postoperative outcomes.

Increasing evidence indicates that a seroma is not merely a passive accumulation of serous fluid but rather an exudative process driven by acute inflammation and tissue injury [17]. Postoperative cytokine release, increased vascular permeability, and enhanced fibrinolytic activity contribute to the formation and persistence of seroma fluid [18,19]. Fibrin organization, fibroblast activation, angiogenesis, and extracellular matrix remodeling are important contributors to postoperative seroma dynamics and tissue repair. Although various surgical techniques, energy-based devices, and pharmacological agents have been investigated to reduce seroma formation, the results remain inconsistent across studies [20,21]. In this context, the present study evaluated the effects of porcine dermal collagen (PDC), tranexamic acid (TXA), and thymoquinone (TQ) using integrated histopathological, immunohistochemical, and molecular analyses.

The results demonstrated that all treatment groups were associated with reduced seroma volume compared to the controls, with more pronounced reductions observed in the TQ and TXA groups, whereas PDC showed a comparatively moderate effect. These macroscopic findings were supported by distinct region-dependent histopathological and molecular patterns across the treatment groups.

PDC treatment primarily induces extracellular matrix-related changes. Increased collagen deposition and fibrin formation, particularly in the thoracic tissue, were accompanied by elevated VEGF and TGF-β1 expression. However, these increases were not uniformly observed across all regions and time points, indicating region-dependent remodeling patterns. The observed increase in p53 expression may reflect regulated cellular turnover and tissue remodeling activity; however, p53 activation may also be associated with cellular stress responses and injury-related signaling pathways following surgical trauma. The moderate and localized increase in p53 expression may reflect regulated cellular turnover and tissue remodeling. However, p53 activation may also be associated with cellular stress responses, apoptosis regulation, and injury-related signaling pathways following surgical trauma.

In contrast, TXA exhibits a more selective biological profile. VEGF expression was consistently elevated, particularly in the axillary and thoracic tissues, whereas TGF-β1 expression and collagen accumulation were comparatively limited. Given the antifibrinolytic properties of TXA, this pattern likely reflects the modulation of postoperative fibrinolytic activity, leading to reduced seroma formation without extensive extracellular matrix remodeling. The absence of marked changes in p53 expression further supports the notion of minimal activation of stress-related cellular pathways during TXA treatment. These findings are generally consistent with those of previous clinical studies reporting reduced postoperative drainage volume, hematoma formation, and seroma incidence following topical or systemic TXA administration in breast surgery procedures. However, the reported clinical efficacy of TXA remains somewhat heterogeneous across studies, likely due to differences in surgical techniques, administration routes, dosing protocols, and patient characteristics. Unlike many previous clinical investigations that primarily focused on postoperative drainage outcomes, the present study additionally incorporated histopathological, immunohistochemical, and molecular analyses, thereby providing complementary mechanistic insights into tissue remodeling responses associated with TXA treatment.

Among the tested interventions, TQ was associated with broader and more pronounced histopathological and molecular alterations in multiple anatomical regions. Histopathological analyses revealed increased vascular proliferation and collagen deposition, particularly in the thoracic tissue, accompanied by a significant upregulation of VEGF, FGF2, and TGF-β1 gene expression. Furthermore, the increased p53 expression observed in the skin and thoracic tissues may indicate the activation of tissue remodeling-associated cellular responses, including regulated repair mechanisms, apoptosis control, and stress-related signaling pathways during postoperative healing. However, these findings should be interpreted cautiously, as increased inflammatory infiltration, vascular proliferation, edema, and fibrin deposition may reflect not only active tissue repair processes but also prolonged inflammatory activation and enhanced fibrotic remodeling, depending on the biological context. Therefore, TQ-associated findings likely represent complex postoperative tissue remodeling responses rather than uniformly beneficial healing effects. However, the present experimental design does not allow for definitive discrimination between adaptive regenerative signaling and stress-associated p53 activation. Additional mechanistic analyses involving apoptosis, oxidative stress, and proliferation-associated markers are necessary to further clarify the biological significance of p53 signaling in postoperative tissue remodeling. The concordance between histopathological, immunohistochemical, and molecular findings suggests that TQ may influence angiogenic, inflammatory, and tissue remodeling pathways in this experimental setting.

The histopathological findings of the present study are consistent with those of previous studies. In a PDC-based model, reduced seroma formation has been associated with increased vascular proliferation, granulation tissue formation, and congestion. Similar effects have been reported with various sclerosing agents, including fibrin glue [22], Mytilus edulis protein [23], and albumin–glutaraldehyde-based sealants [24], supporting the role of extracellular matrix modulation in seroma prevention.

The inflammatory nature of seroma formation has also been supported by studies investigating pharmacological modulation of postoperative inflammation. Steroids and nonsteroidal anti-inflammatory drugs (NSAIDs) have been shown to reduce seroma formation, although their relative efficacy remains variable [25]. TXA, through its antifibrinolytic mechanism, has been associated with decreased seroma formation in several studies; however, conflicting findings have also been reported [26]. In addition, experimental approaches using mesenchymal stem cells [27], collagen-based fibrin sealants [28], and platelet-rich plasma (PRP) [29] have demonstrated increased fibroblast activity, neovascularization, and reduced seroma formation. The findings of the present study are in agreement with these reports, highlighting the importance of coordinated angiogenic and remodeling responses in the regulation of postoperative fluid accumulation. Fibrin organization, fibroblast activation, angiogenesis, and extracellular matrix remodeling appear to be closely interconnected components of postoperative seroma biology and wound healing. Fibrin deposition may contribute to the temporary stabilization of the surgical dead space and local wound architecture, whereas impaired fibrin maturation and excessive fibrinolytic activity may facilitate persistent postoperative fluid accumulation. In addition, fibroblast-mediated extracellular matrix remodeling plays a critical role in collagen deposition, granulation tissue formation, and structural stabilization during tissue repair. Angiogenic signaling pathways, particularly those associated with VEGF and FGF2, are essential for vascular remodeling, inflammatory cell recruitment, nutrient delivery, and tissue regeneration. However, excessive vascular permeability and dysregulated angiogenic responses may contribute to persistent seroma formation and prolonged inflammatory activation following surgical injury.

An important observation of this study was the consistent responsiveness of thoracic tissue across all treatment modalities. This finding suggests that certain anatomical regions may exhibit increased sensitivity to surgical trauma and therapeutic interventions, emphasizing the importance of region-specific biological responses in postoperative tissue remodeling.

Although the present findings provide important experimental insights into postoperative seroma formation and tissue remodeling, the translational interpretation of these results should be approached cautiously. The current study was performed in a controlled rat mastectomy model using a relatively limited number of animals and a single, postoperative endpoint. Furthermore, rodent wound healing dynamics and inflammatory responses differ substantially from those observed in postoperative human patients who underwent breast surgery. In addition, several clinically relevant variables influencing postoperative wound healing and seroma formation in humans, including patient age, obesity, diabetes, smoking status, immunological heterogeneity, chemotherapy, radiotherapy, hormonal therapies, and tumor-associated microenvironmental factors, cannot be fully reproduced under standardized experimental conditions. Therefore, although thymoquinone, tranexamic acid, and porcine dermal collagen demonstrated biologically relevant effects in this experimental setting, additional validation through larger preclinical studies and clinical investigations is necessary before definitive therapeutic or translational conclusions can be established.

Taken together, the results of this study demonstrate that TXA primarily exerts a targeted angiogenic effect with limited matrix remodeling, PDC promotes structural stabilization through extracellular matrix deposition, and TQ is associated with broader angiogenic, inflammatory, and tissue remodeling responses within this experimental rat model. These differential biological responses provide mechanistic insights into the regulation of postoperative seroma formation and may contribute to future experimental investigations aimed at optimizing postoperative wound-healing strategies.

## 5. Conclusions

This study demonstrated that porcine dermal collagen (PDC), tranexamic acid (TXA), and thymoquinone (TQ) differentially modulate postoperative seroma formation via distinct histopathological and molecular mechanisms. TXA appeared to exert a predominantly anti-seroma effect, likely associated with the modulation of fibrinolytic activity, whereas PDC promoted extracellular matrix stabilization through enhanced collagen deposition and fibrin organization. In contrast, TQ was associated with broader inflammatory, angiogenic, and tissue remodeling responses in this experimental rat model. However, these findings should be interpreted cautiously, as the increased inflammatory infiltration, edema, vascular proliferation, and fibrin deposition observed in the TQ group may also reflect enhanced tissue reactivity, persistent inflammatory activation, or active remodeling processes, rather than uniformly beneficial healing effects. Therefore, although TQ influenced postoperative tissue remodeling and seroma-associated responses in this model, further mechanistic and clinical studies are required before translational conclusions can be drawn from these results.

Several limitations should be considered when interpreting the present study’s findings. Although the rat mastectomy model provides a controlled and reproducible platform for investigating postoperative seroma formation and tissue remodeling, direct extrapolation to human breast surgery remains limited because of important differences in anatomy, immune response, wound healing kinetics, and overall clinical complexity. Moreover, clinically relevant variables such as comorbidities, adjuvant therapies, and tumor-associated microenvironmental factors cannot be fully reproduced under standardized experimental conditions. Despite the use of postoperative analgesia and standardized animal welfare protocols, perioperative stress and pain responses may still influence inflammatory signaling and tissue remodeling dynamics. Additional limitations include the relatively small sample size, evaluation at a single postoperative time point, absence of dose–response and systemic safety analyses, and lack of cell-type-specific inflammatory characterization. Furthermore, the study primarily relied on semi-quantitative histopathological and immunohistochemical assessments without digital morphometric quantification or broader mechanistic pathway analysis. Finally, representative histopathological images were selected to illustrate the principal findings and therefore do not comprehensively cover all experimental groups, anatomical regions, and staining modalities. Future studies integrating larger cohorts, longitudinal analyses, advanced digital imaging approaches, and expanded molecular characterization may provide deeper mechanistic insights into postoperative seroma biology and tissue remodeling processes.

## Figures and Tables

**Table 1:** Comparative histopathological evaluation of PDC, TXA, and TQ treatments in different anatomical regions. * *p* < 0.05; ** *p* < 0.01; *** *p* < 0.001; **** *p* < 0.0001.

TXA	Inflammatory Cell Infiltration								
	**Axilla**			**Skin**			**Thorax**		
	C_0_	C_14_	T	C_0_	C_14_	T	C_0_	C_14_	T
	0.2 ± 0.42	2.25 ± 0.70 ****	3.25 ± 1.03 *****	0.5 ± 0.52	2.8 ± 0.63 ****	2.2 ± 0.63 ****	0.1 ± 0.31	2.8 ± 0.78 ****	2.77 ± 0.44 ****
				Edema					
	**Axilla**			**Skin**			**Thorax**		
	C_0_	C_14_	T	C_0_	C_14_	T	C_0_	C_14_	T
	0.5 ± 0.52	2.11 ± 0.60 ****	2 ± 0.81 ****	0.7 ± 0.48	2.1 ± 0.56 ****	2 ± 0.66 ****	0.1 ± 0.3	2.2 ± 0.63 ****	2.4 ± 0.84 ****
	Vascular Congestion/Proliferation								
	**Axilla**			**Skin**			**Thorax**		
	C_0_	C_14_	T	C_0_	C_14_	T	C_0_	C_14_	T
	1.4 ± 0.69	1.66 ± 0.70	2.4 ± 0.84 *	0.6 ± 0.84	1.9 ± 0.73 **	2.1 ± 0.73 ***	0.1 ± 0.31	2 ± 0.94 ****	2 ± 0.81 ****
	Collagen Accumulation								
	**Axilla**			**Skin**			**Thorax**		
	C_0_	C_14_	T	C_0_	C_14_	T	C_0_	C_14_	T
	0.77 ± 0.44	2.44 ± 1.01 ****	1.6 ± 0.84	1.1 ± 0.31	2.6 ± 0.69 ****	1.8 ± 0.78	0.6 ± 0.51	1.6 ± 0.96 *	0.9 ± 0.73
	Fibrin Formation								
	**Axilla**			**Skin**			**Thorax**		
	C_0_	C_14_	T	C_0_	C_14_	T	C_0_	C_14_	T
	0.3 ± 0.48	1.44 ± 0.52 *	2.6 ± 1.26 ****	0.1 ± 0.1	0.5 ± 0.52	0.9 ± 0.99	0.2 ± 0.63	1.1 ± 0.73	1.1 ± 0.99
TQ	Inflammatory Cell Infiltration								
	**Axilla**			**Skin**			**Thorax**		
	C_0_	C_14_	T	C_0_	C_14_	T	C_0_	C_14_	T
	0.2 ± 0.42	2.25 ± 0.70 ****	3.3 ± 0.86 ****	0.5 ± 0.52	2.8 ± 0.63 ****	3 ± 0.47 ****	0.1 ± 0.31	2.8 ± 0.78 ****	3.2 ± 0.91 ****
	Edema								
	**Axilla**			**Skin**			**Thorax**		
	C_0_	C_14_	T	C_0_	C_14_	T	C_0_	C_14_	T
	0.5 ± 0.52	2.11 ± 0.60 ****	2.2 ± 0.78 ****	0.7 ± 0.48	2.1 ± 0.56 ****	2.6 ± 0.69 ****	0.1 ± 0.3	2.2 ± 0.63 ****	2.7 ± 0.67 ****
	Vascular Congestion/Proliferation								
	**Axilla**			**Skin**			**Thorax**		
	C_0_	C_14_	T	C_0_	C_14_	T	C_0_	C_14_	T
	1.4 ± 0.69	1.66 ± 0.70	2.3 ± 0.82	0.6 ± 0.84	1.9 ± 0.73 **	2.6 ± 0.69 ****	0.1 ± 0.31	2 ± 0.94 ****	2.6 ± 0.84 ****
	Collagen Accumulation								
	**Axilla**			**Skin**			**Thorax**		
	C_0_	C_14_	T	C_0_	C_14_	T	C_0_	C_14_	T
	0.77 ± 0.44	2.44 ± 1.01 ***	3 ± 0.94 ****	1.1 ± 0.31	2.6 ± 0.69 ***	2.9 ± 0.99 ****	0.6 ± 0.51	1.6 ± 0.96	2.8 ± 1.22 ****
	Fibrin Formation								
	**Axilla**			**Skin**			**Thorax**		
	C_0_	C_14_	T	C_0_	C_14_	T	C_0_	C_14_	T
	0.3 ± 0.48	1.44 ± 0.52 *	2.5 ± 1.08 ****	0.1 ± 0.1	0.5 ± 0.52	1.8 ± 0.63 ****	0.2 ± 0.63	1.1 ± 0.73 *	1.9 ± 0.99 ****
PDC	Inflammatory Cell Infiltration								
	**Axilla**			**Skin**			**Thorax**		
	C_0_	C_14_	T	C_0_	C_14_	T	C_0_	C_14_	T
	0.2 ± 0.42	2.25 ± 0.70 ****	3 ± 0.7 ****	0.5 ± 0.52	2.8 ± 0.63 ****	2.6 ± 0.96 ****	0.1 ± 0.31	2.8 ± 0.78 ****	3.3 ± 0.82 ****
	Edema								
	**Axilla**			**Skin**			**Thorax**		
	C_0_	C_14_	T	C_0_	C_14_	T	C_0_	C_14_	T
	0.5 ± 0.52	2.11 ± 0.60 ****	2.4 ± 0.51 ****	0.7 ± 0.48	2.1 ± 0.56 ****	1.9 ± 0.73 ****	0.1 ± 0.3	2.2 ± 0.63 ****	2.8 ± 0.42 ****
	Vascular Congestion/Proliferation								
	**Axilla**			**Skin**			**Thorax**		
	C_0_	C_14_	T	C_0_	C_14_	T	C_0_	C_14_	T
	1.4 ± 0.69	1.66 ± 0.70	2.3 ± 0.67 *	0.6 ± 0.84	1.9 ± 0.73 ***	1.9 ± 0.73 ***	0.1 ± 0.31	2 ± 0.94 ****	2.6 ± 0.69 ****
	Collagen Accumulation								
	**Axilla**			**Skin**			**Thorax**		
	C_0_	C_14_	T	C_0_	C_14_	T	C_0_	C_14_	T
	0.77 ± 0.44	2.44 ± 1.01 ****	2.3 ± 1.05 ***	1.1 ± 0.31	2.6 ± 0.69 ***	3.1 ± 0.87 ****	0.6 ± 0.51	1.6 ± 0.96 *	2.7 ± 0.82 ****
	Fibrin Formation								
	**Axilla**			**Skin**			**Thorax**		
	C_0_	C_14_	T	C_0_	C_14_	T	C_0_	C_14_	T
	0.3 ± 0.48	1.44 ± 0.52 *	2.2 ± 1.13 ****	0.1 ± 0.1	0.5 ± 0.52	1.4 ± 1.17 **	0.2 ± 0.63	1.1 ± 0.73	2.3 ± 1.15 ****

C_0_: Baseline tissue samples collected intraoperatively before treatment application; C_14_/T_14_: Postoperative day 14 tissue samples used for comparative histopathological analyses.

## Data Availability

Data supporting the findings of this study are available from the corresponding author upon reasonable request.

## Supplementary Materials

The following supporting information can be downloaded at https://www.mdpi.com/article/10.3390/medicina62071228/s1, Figure S1: Representative histopathological and immunohistochemical microphotographs of postoperative tissues obtained from the control group following modified radical mastectomy and axillary dissection. Images are selected representative photomicrographs illustrating key qualitative histopathological and immunohistochemical findings. Complete quantitative histopathological and immunohistochemical data for all groups, anatomical regions, and markers are presented in Table S1 and Figures S1–S3. (a) H&E staining of control group axillary tissue demonstrating baseline postoperative inflammatory cell infiltration and edema (×100). (b) H&E staining of control group skin tissue demonstrating postoperative wound architecture with preserved epidermal and adnexal structures (×100). (c) H&E staining of control group thoracic tissue showing inflammatory cell infiltration, edema, and vascular congestion/proliferation (×100). (d) Representative immunohistochemical staining from control group tissue sections showing weak-to-moderate marker positivity (×400). (e) Representative immunohistochemical staining from control group tissue demonstrating focal stromal and perivascular positive staining (×400). (f) Representative immunohistochemical staining from control group tissue showing scattered positive cells within fibroblast-rich connective tissue regions (×400); Figure S2: Representative immunohistochemical staining patterns of FGF2, VEGF, TGF-β1, and p53 expression in postoperative control group tissues following modified radical mastectomy and axillary dissection. Images shown are selected representative photomicrographs illustrating baseline postoperative immunohistochemical expression patterns in the control group. Complete quantitative immunohistochemical analyses for all groups, anatomical regions, and markers are presented in Figures S1–S3 and Table S1. (a–c) FGF2 immunohistochemistry (×400): (a) Axillary tissue demonstrating weak stromal and endothelial cytoplasmic staining. (b) Skin tissue showing mild cytoplasmic positivity within dermal stromal regions. (c) Thoracic tissue demonstrating low-to-moderate stromal cytoplasmic immunoreactivity. (d–f) VEGF immunohistochemistry (×400): (d) Axillary tissue showing weak endothelial and perivascular staining. (e) Skin tissue demonstrating mild vascular and stromal VEGF positivity. (f) Thoracic tissue showing limited endothelial cytoplasmic immunoreactivity. (g–i) TGF-β1 immunohistochemistry (×400): (g) Axillary tissue demonstrating weak baseline cytoplasmic staining. (h) Skin tissue showing focal stromal and connective tissue positivity. (i) Thoracic tissue demonstrating mild diffuse cytoplasmic immunoreactivity. (j–l) p53 immunohistochemistry (×400): (j) Axillary tissue showing weak nuclear p53 positivity. (k) Skin tissue demonstrating scattered nuclear immunopositive cells. (l) Thoracic tissue showing minimal baseline nuclear staining; Figure S3: Representative histopathological microphotographs of postoperative tissues obtained from the porcine dermal collagen (PDC)-treated group following modified radical mastectomy and axillary dissection. Images shown are selected representative photomicrographs illustrating qualitative histopathological findings in the PDC-treated group. Complete quantitative histopathological analyses for all groups and anatomical regions are presented in Table S1. (a–c) Hematoxylineosin (H&E) staining (×100). (a) Axillary tissue demonstrating prominent inflammatory cell infiltration, edema, vascular congestion/proliferation, and fibrin deposition. (b) Skin tissue showing marked inflammatory infiltration and postoperative tissue remodeling within the dermal layer. (c) Thoracic tissue demonstrating inflammatory infiltration, edema, vascular congestion/proliferation, and fibrin accumulation within the surgical field. (d–f) Masson’s trichrome staining (×200). (d) Axillary tissue showing moderate collagen deposition and extracellular matrix organization. (e) Skin tissue demonstrating dense collagen deposition within dermal connective tissue regions. (f) Thoracic tissue showing dense collagen accumulation and fibrotic extracellular matrix remodeling; Figure S4: Representative immunohistochemical staining patterns of FGF2, VEGF, TGF-β1, and p53 in postoperative tissues obtained from the porcine dermal collagen (PDC)-treated group following modified radical mastectomy and axillary dissection. Images shown are selected representative photomicrographs illustrating qualitative immunohistochemical findings in the PDC-treated group. Complete quantitative immunohistochemical analyses for all groups, regions, and markers are presented in Figures S1–S3 and Table S1. (a–c) FGF2 immunohistochemistry (×400). (a) Axillary tissue showing moderate cytoplasmic positivity in fibroblasts and stromal cells. (b) Skin tissue demonstrating moderate FGF2 expression within dermal stromal regions. (c) Thoracic tissue showing diffuse cytoplasmic FGF2 positivity in fibroblastic and stromal compartments. (d–f) VEGF immunohistochemistry (×400). (d) Axillary tissue demonstrating marked VEGF positivity within endothelial and perivascular regions. (e) Skin tissue showing increased VEGF-associated endothelial and stromal staining. (f) Thoracic tissue demonstrating intense VEGF immunoreactivity associated with vascular proliferation and stromal activation. (g–i) TGF-β1 immunohistochemistry (×400). (g) Axillary tissue showing increased cytoplasmic TGF-β1 positivity in fibroblasts and inflammatory cells. (h) Skin tissue demonstrating strong TGF-β1 expression within dermal connective tissue regions. (i) Thoracic tissue exhibiting prominent TGF-β1 positivity associated with extracellular matrix remodeling and fibroblast activation. (j–l) p53 immunohistochemistry (×400). (j) Axillary tissue demonstrating marked nuclear p53 positivity. (k) Skin tissue showing relatively limited p53 immunoreactivity with focal nuclear staining. (l) Thoracic tissue demonstrating increased nuclear p53 positivity within stromal and inflammatory cellular components; Figure S5: Representative histopathological and immunohistochemical findings in the tranexamic acid (TXA)-treated group following modified radical mastectomy and axillary dissection. Images shown are selected representative photomicrographs illustrating qualitative histopatholog-ical and immunohistochemical findings in the TXA-treated group. Complete quantitative histopathological and immunohistochemical analyses for all groups, regions, and markers are present-ed in Table S1 and Figures S1–S3. (a–c) Hematoxylin and eosin (H&E) staining (×100). (a) Axillary tissue demonstrating prominent inflammatory cell infiltration, edema, vascular congestion/proliferation, and fibrin deposition. (b) Skin tissue showing prominent inflammatory infiltration, edema, and vascular congestion/proliferation within the dermal connective tissue. (c) Thoracic tissue demonstrating inflammatory infiltration, edema, and vascular congestion/proliferation within the surgical field. (d–f) Masson’s trichrome staining (×200). (d) Axillary tissue demonstrating moderate collagen deposition within dermal connective tissue regions. (e) Skin tissue demonstrating moderate collagen deposition within dermal connective tissue regions. (f) Thoracic tissue showing limited/sparse collagen deposition, consistent with a relatively low tissue-remodeling profile; Figure S6: Representative immunohistochemical staining patterns in the tranexamic acid (TXA)-treated group following modified radical mastectomy and axillary dissection (×400). Images shown are representative photomicrographs illustrating qualitative immunohistochemical findings in the TXA-treated group. Complete quantitative H-score analyses for all markers, anatomical regions, and experimental groups are presented in Figures S1–S3 and Table S1. (a–c) FGF2 immunohistochemistry. (a) Axillary tissue showing weak positive staining without a significant increase, with mild cytoplasmic positivity in fibroblasts and stromal cells. (b) Skin tissue demonstrating increased cytoplasmic FGF2 positivity in fibroblasts and stromal cells. (c) Thoracic tissue showing weak baseline-level FGF2 staining. (d–f) VEGF immunohistochemistry. (d) Axillary tissue demonstrating marked and intense VEGF positivity, predominantly localized in endothelial and perivascular regions. (e) Skin tissue showing moderate VEGF immunoreactivity with cytoplasmic positivity in endothelial and perivascular cells. (f) Thoracic tissue demonstrating marked VEGF positivity associated with vascular structures and stromal regions. (g–i) TGF-β1 immunohistochemistry. (g) Axillary tissue showing moderate cytoplasmic TGF-β1 positivity in fibroblasts and inflammatory cells. (h) Skin tissue demonstrating marked TGF-β1 immunoreactivity with increased staining intensity in stromal and inflammatory regions. (i) Thoracic tissue showing intense TGF-β1 positivity within fibroblast-rich stromal areas. (j–l) p53 immunohistochemistry. (j) Axillary tissue demonstrating slightly increased nuclear p53 positivity. (k) Skin tissue showing stable baseline-level p53 staining without marked increase. (l) Thoracic tissue demonstrating weak baseline-level nuclear p53 immunoreactivity; Figure S7: Representative histopathological findings in the thymoquinone (TQ)-treated group following modified radical mastectomy and axillary dissection. (a) H&E staining, TQ group, axillary tissue (×100). Prominent inflammatory cell infiltration, edema, vascular congestion/proliferation, and fibrin deposition. (b) H&E staining, TQ group, skin tissue (×100). Prominent inflammatory cell infiltration, edema, and vascular congestion/proliferation. (c) H&E staining, TQ group, thoracic tissue (×100). Prominent inflammatory cell infiltration, edema, and vascular congestion/proliferation. (d) Masson’s trichrome staining, TQ group, axillary tissue (×200). Dense collagen deposition. (e) Masson’s trichrome staining, TQ group, skin tissue (×200). Dense collagen deposition. (f) Masson’s trichrome staining, TQ group, thoracic tissue (×200). Dense collagen deposition; Figure S8: Representative immunohistochemical findings in the thymoquinone (TQ)-treated group following modified radical mastectomy and axillary dissection (×400). (a) FGF2 immunohistochemistry, TQ group, axillary tissue (×400). Increased positive staining, with cytoplasmic positivity in fibroblasts and stromal cells. (b) FGF2 immunohistochemistry, TQ group, skin tissue (×400). Increased positive staining, with cytoplasmic positivity in fibroblasts and stromal cells. (c) FGF2 immunohistochemistry, TQ group, thoracic tissue (×400). The most prominent positive staining observed in the study, with cytoplasmic positivity in fibroblasts and stromal cells. (d) VEGF immunohistochemistry, TQ group, axillary tissue (×400). Increased positive staining, with cytoplasmic positivity in endothelial and perivascular areas. (e) VEGF immunohistochemistry, TQ group, skin tissue (×400). The most intense positive staining (the highest expression observed in the study), with cytoplasmic positivity in endothelial and perivascular areas. (f) VEGF immunohistochemistry, TQ group, thoracic tissue (×400). The most intense positive staining (the highest expression observed in the study), with cytoplasmic positivity in endothelial and perivascular areas. (g) TGF-β1 immunohistochemistry, TQ group, axillary tissue (×400). Increased pos-itive staining, with cytoplasmic positivity in fibroblasts and inflammatory cells. (h) TGF-β1 immunohistochemistry, TQ group, skin tissue (×400). Marked, intense positive staining, with cytoplasmic positivity in fibroblasts and inflammatory cells. (i) TGF-β1 immunohistochemistry, TQ group, thoracic tissue (×400). Marked, intense positive staining, with cytoplasmic positivity in fibroblasts and inflammatory cells. (j) p53 immunohistochemistry, TQ group, axillary tissue (×400). Slightly increased positive staining, with nuclear positivity. (k) p53 immunohistochemistry, TQ group, skin tissue (×400). Stable, baseline-level staining (no notable change), with nuclear positivity. (l) p53 immunohistochemistry, TQ group, thoracic tissue (×400). Stable, baseline-level staining (no notable change), with nuclear positivity; Figure S9: Workflow after experiment; Table S1: Absolute seroma volume measurements in experimental groups on postoperative day 14.

## Abbreviations

PDC	Porcine Dermal Collagen
TXA	Tranexamic Acid
TQ	Thymoquinone
FGF2	Fibroblast Growth Factor 2
VEGF	Vascular Endothelial Growth Factor
TGF-β1	Transforming Growth Factor Beta 1
