# Supplementary material for: Comparative Effects of Thymoquinone, Tranexamic Acid, and Porcine Dermal Collagen on Seroma Formation and Tissue Remodeling After Mastectomy in a Rat Model
Source: Medicina (Kaunas). 2026 Jun 24;62(7):1228. doi: 10.3390/medicina62071228 (PMC13413573; doi:10.3390/medicina62071228)
Supplement: Supplementary file 1 [file medicina-62-01228-s001.zip › medicina-4329314-supplementary.pdf]

Supplementary Materials

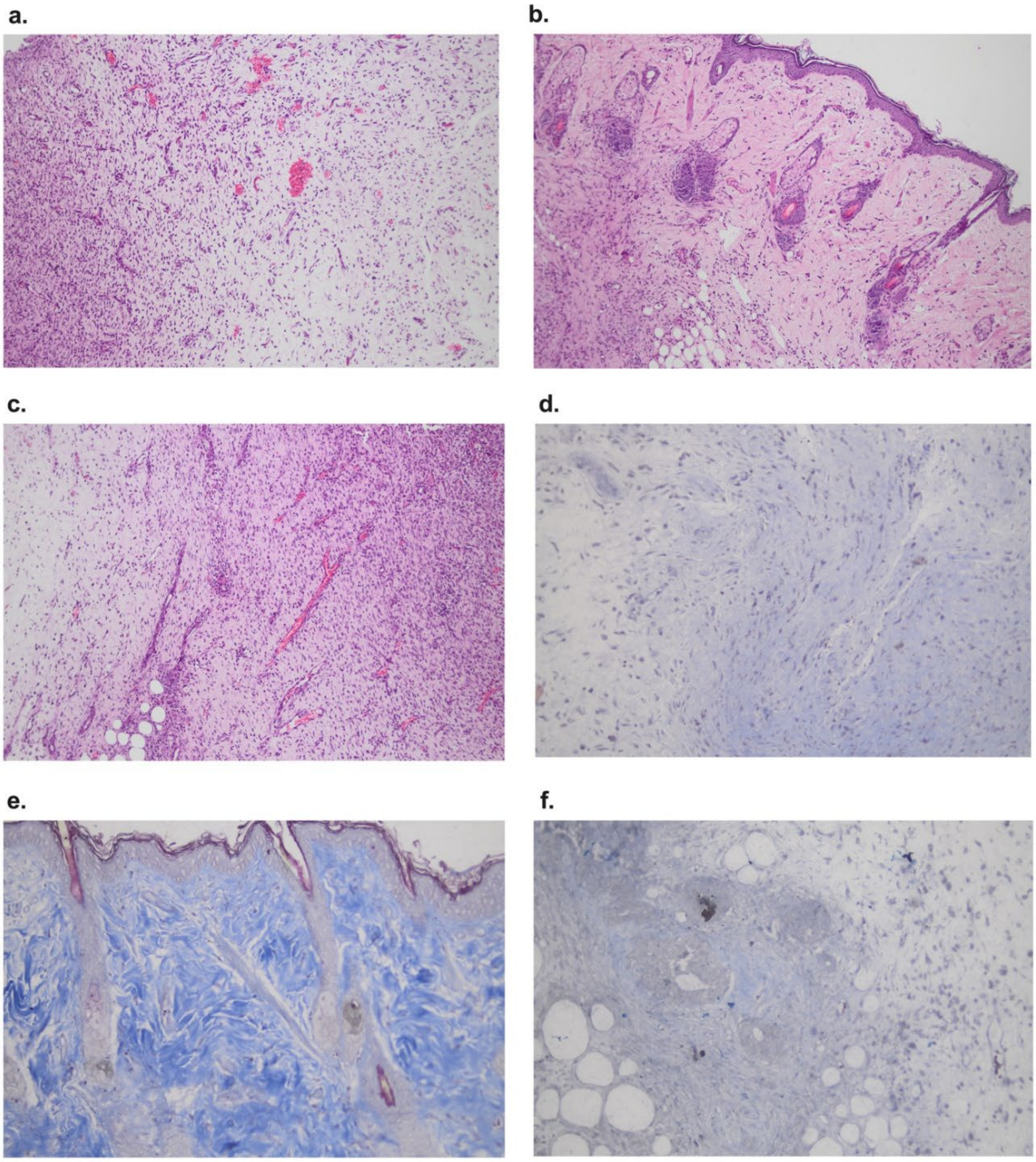

Figure S1. Representative histopathological and immunohistochemical microphotographs of postoperative tissues obtained from the control group following modified radical mastectomy and axillary dissection. Images are selected representative photomicrographs illustrating key qualitative histopathological and immunohistochemical findings. Complete quantitative histopathological and immunohistochemical data for all groups, anatomical regions, and markers are presented in Table S1 and Figures S1–S3. (a) H&E staining of control group axillary tissue demonstrating baseline postoperative inflammatory cell infiltration and edema (×100). (b) H&E staining of control group skin tissue demonstrating postoperative wound architecture with preserved epidermal and adnexal structures (×100). (c) H&E staining of control group thoracic tissue showing inflammatory cell infiltration, edema, and vascular congestion/proliferation (×100). (d) Representative immunohistochemical staining from control group tissue sections showing weak-to-moderate marker positivity (×400). (e) Representative immunohistochemical staining from control group tissue demonstrating focal stromal and perivascular positive staining (×400). (f) Representative immunohistochemical staining from control group tissue showing scattered positive cells within fibroblast-rich connective tissue regions (×400).

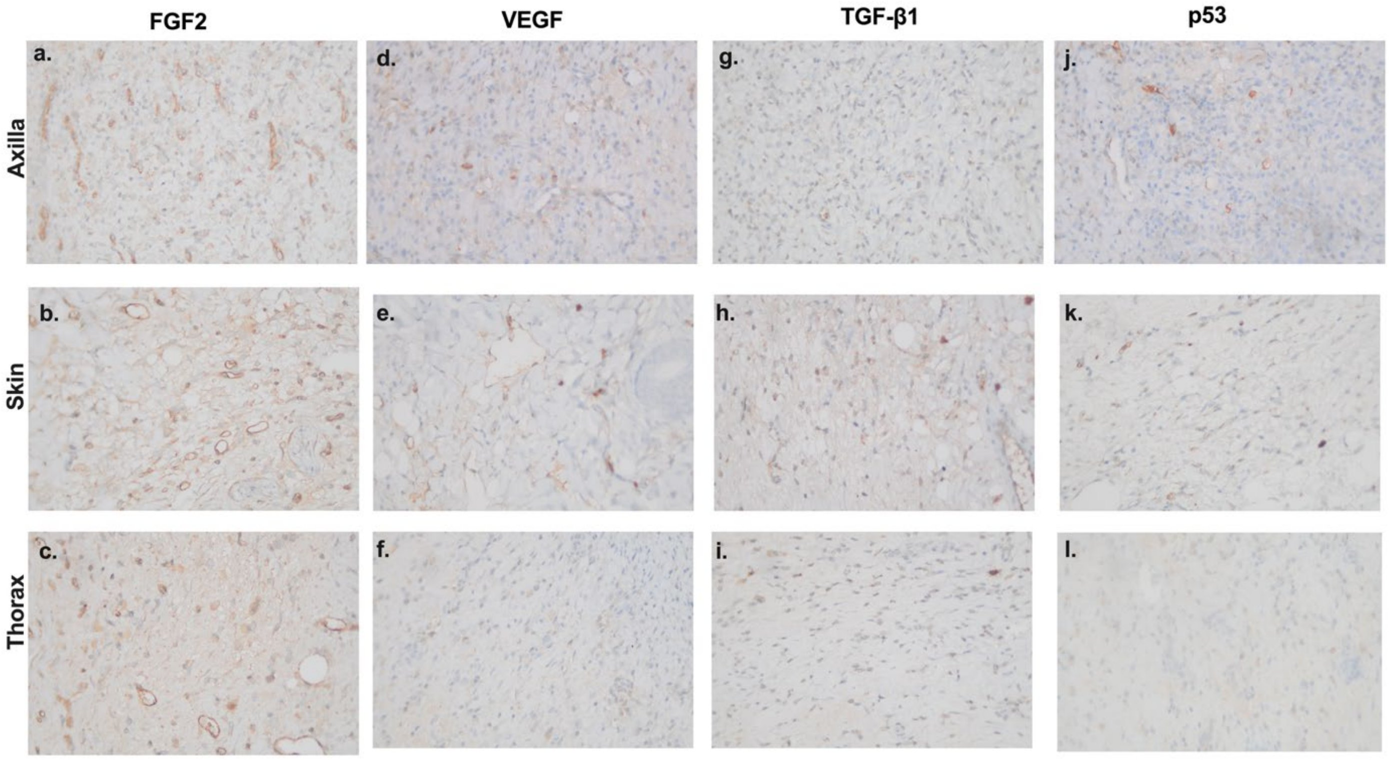

Figure S2. Representative immunohistochemical staining patterns of FGF2, VEGF, TGF-β1, and p53 expression in postoperative control group tissues following modified radical mastectomy and axillary dissection. Images shown are selected representative photomicrographs illustrating baseline postoperative immunohistochemical expression patterns in the control group. Complete quantitative immunohistochemical analyses for all groups, anatomical regions, and markers are presented in Figures S1–S3 and Table S1. (a-c) FGF2 immunohistochemistry (×400): (a) Axillary tissue demonstrating weak stromal and endothelial cytoplasmic staining. (b) Skin tissue showing mild cytoplasmic positivity within dermal stromal regions. (c) Thoracic tissue demonstrating low-to-moderate stromal cytoplasmic immunoreactivity. (d-f) VEGF immunohistochemistry (×400): (d) Axillary tissue showing weak endothelial and perivascular staining. (e) Skin tissue demonstrating mild vascular and stromal VEGF positivity. (f) Thoracic tissue showing limited endothelial cytoplasmic immunoreactivity. (g-i) TGF-β1 immunohistochemistry (×400): (g) Axillary tissue demonstrating weak baseline cytoplasmic staining. (h) Skin tissue showing focal stromal and connective tissue positivity. (i) Thoracic tissue demonstrating mild diffuse cytoplasmic immunoreactivity. (j-l) p53 immunohistochemistry (×400): (j) Axillary tissue showing weak nuclear p53 positivity. (k) Skin tissue demonstrating scattered nuclear immunopositive cells. (l) Thoracic tissue showing minimal baseline nuclear staining.

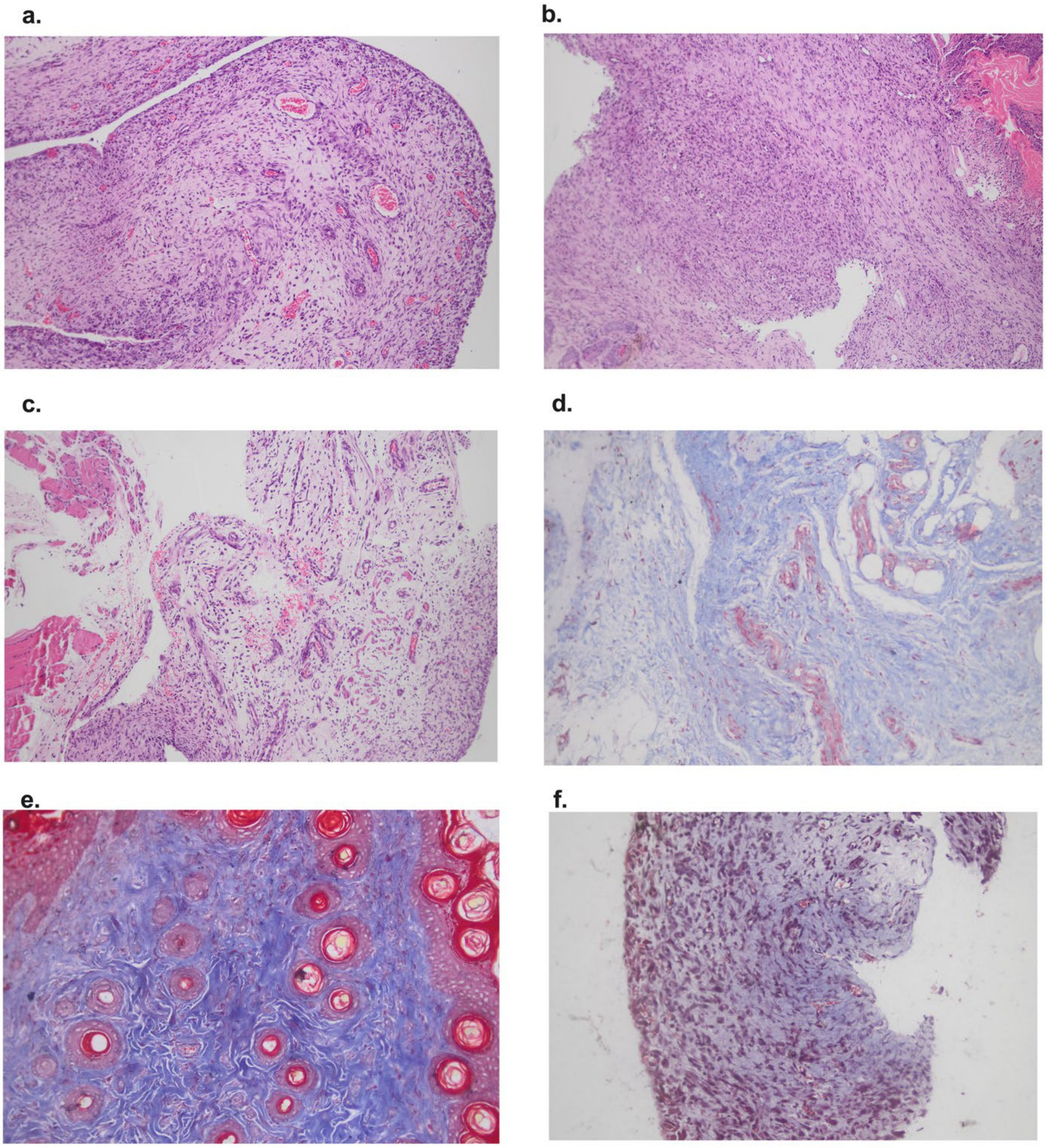

Figure S3. Representative histopathological microphotographs of postoperative tissues obtained from the porcine dermal collagen (PDC)-treated group following modified radical mastectomy and axillary dissection. Images shown are selected representative photomicrographs illustrating qualitative histopathological findings in the PDC-treated group. Complete quantitative histopathological analyses for all groups and anatomical regions are presented in Table S1. (a-c) Hematoxylin-eosin (H&E) staining (×100). (a) Axillary tissue demonstrating prominent inflammatory cell infiltration, edema, vascular congestion/proliferation, and fibrin deposition. (b) Skin tissue showing marked inflammatory infiltration and postoperative tissue remodeling within the dermal layer. (c) Thoracic tissue demonstrating inflammatory infiltration, edema, vascular congestion/proliferation, and fibrin accumulation within the surgical field. (d-f) Masson's trichrome staining (×200). (d) Axillary tissue showing moderate collagen deposition and extracellular matrix organization. (e) Skin tissue demonstrating dense collagen deposition within dermal connective tissue regions. (f) Thoracic tissue showing dense collagen accumulation and fibrotic extracellular matrix remodeling.

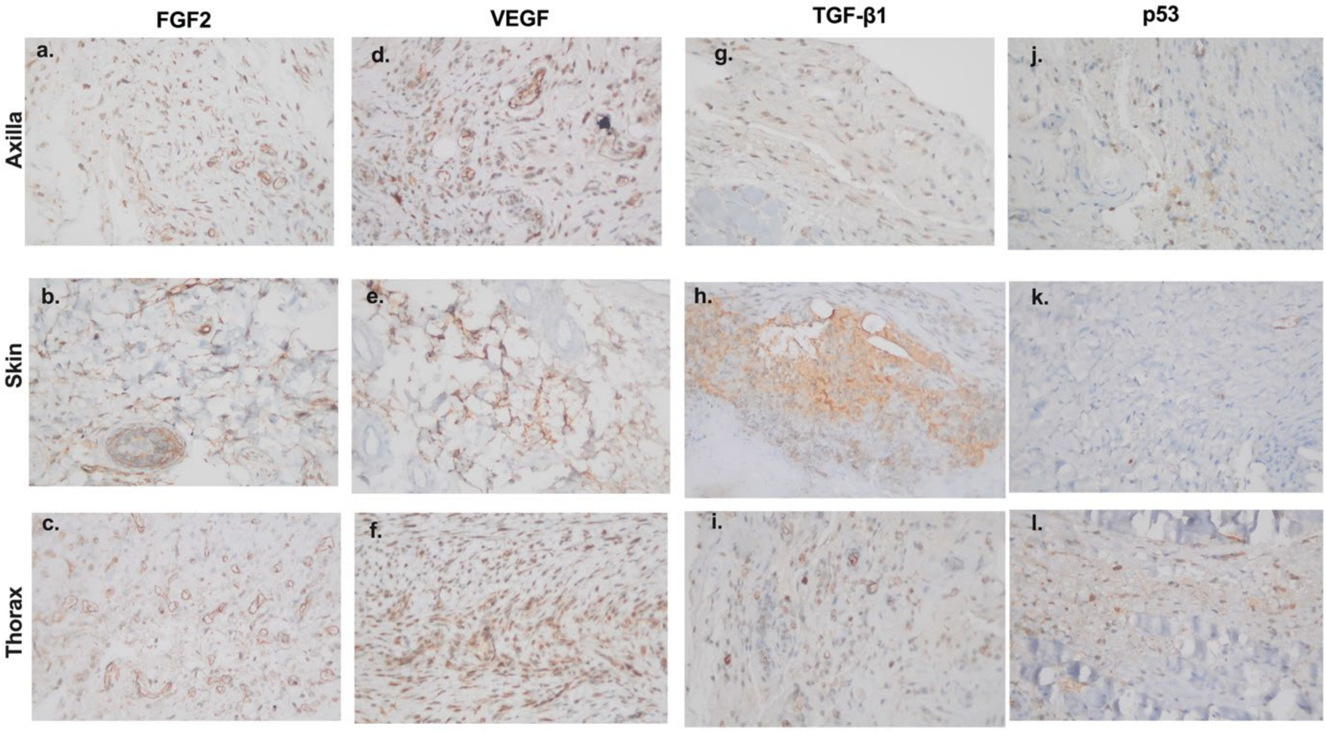

Figure S4. Representative immunohistochemical staining patterns of FGF2, VEGF, TGF-β1, and p53 in postoperative tissues obtained from the porcine dermal collagen (PDC)-treated group following modified radical mastectomy and axillary dissection. Images shown are selected representative photomicrographs illustrating qualitative immunohistochemical findings in the PDC-treated group. Complete quantitative immunohistochemical analyses for all groups, regions, and markers are presented in Figures S1–S3 and Table S1. (a-c) FGF2 immunohistochemistry (×400). (a) Axillary tissue showing moderate cytoplasmic positivity in fibroblasts and stromal cells. (b) Skin tissue demonstrating moderate FGF2 expression within dermal stromal regions. (c) Thoracic tissue showing diffuse cytoplasmic FGF2 positivity in fibroblastic and stromal compartments. (d-f) VEGF immunohistochemistry (×400). (d) Axillary tissue demonstrating marked VEGF positivity within endothelial and perivascular regions. (e) Skin tissue showing increased VEGF-associated endothelial and stromal staining. (f) Thoracic tissue demonstrating intense VEGF immunoreactivity associated with vascular proliferation and stromal activation. (g-i) TGF-β1 immunohistochemistry (×400). (g) Axillary tissue showing increased cytoplasmic TGF-β1 positivity in fibroblasts and inflammatory cells. (h) Skin tissue demonstrating strong TGF-β1 expression within dermal connective tissue regions. (i) Thoracic tissue exhibiting prominent TGF-β1 positivity associated with extracellular matrix remodeling and fibroblast activation. (j-l) p53 immunohistochemistry (×400). (j) Axillary tissue demonstrating marked nuclear p53 positivity. (k) Skin tissue showing relatively limited p53 immunoreactivity with focal nuclear staining. (l) Thoracic tissue demonstrating increased nuclear p53 positivity within stromal and inflammatory cellular components.

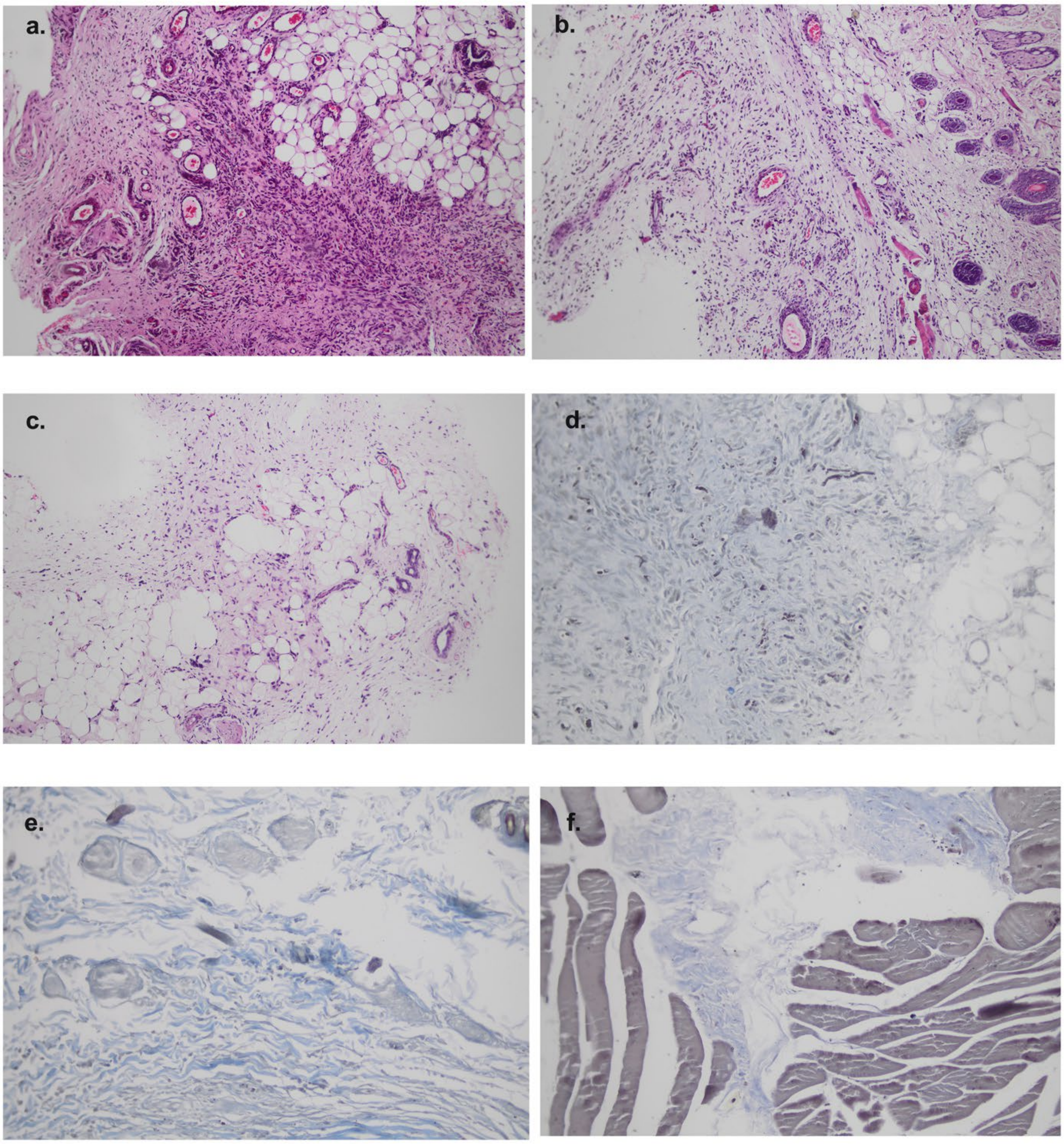

Figure S5. Representative histopathological and immunohistochemical findings in the tranexamic acid (TXA)-treated group following modified radical mastectomy and axillary dissection. Images shown are selected representative photomicrographs illustrating qualitative histopathological and immunohistochemical findings in the TXA-treated group. Complete quantitative histopathological and immunohistochemical analyses for all groups, regions, and markers are presented in Table S1 and Figures S1–S3 . (a-c) Hematoxylin and eosin (H&E) staining (×100). (a) Axillary tissue demonstrating prominent inflammatory cell infiltration, edema, vascular congestion/proliferation, and fibrin deposition. (b) Skin tissue showing prominent inflammatory infiltration, edema, and vascular congestion/proliferation within the dermal connective tissue. (c) Thoracic tissue demonstrating inflammatory infiltration, edema, and vascular congestion/proliferation within the surgical field. (d-f) Masson's trichrome staining (×200). (d) Axillary tissue demonstrating moderate collagen deposition within dermal connective tissue regions. (e) Skin tissue demonstrating moderate collagen deposition within dermal connective tissue regions. (f) Thoracic tissue showing limited/sparse collagen deposition, consistent with a relatively low tissue-remodeling profile.

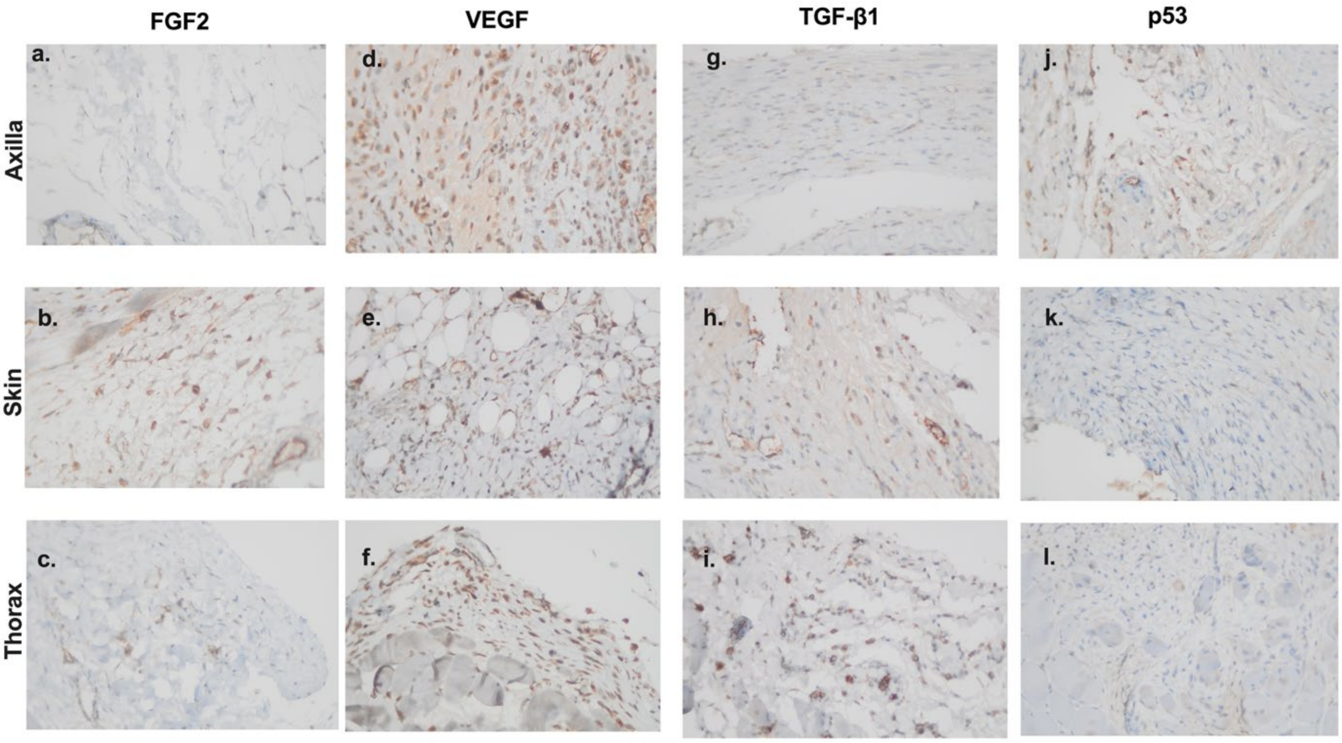

Figure S6. Representative immunohistochemical staining patterns in the tranexamic acid (TXA)-treated group following modified radical mastectomy and axillary dissection (×400). Images shown are representative photomicrographs illustrating qualitative immunohistochemical findings in the TXA-treated group. Complete quantitative H-score analyses for all markers, anatomical regions, and experimental groups are presented in Figures S1–S3 and Table S1. (a-c) FGF2 immunohistochemistry. (a) Axillary tissue showing weak positive staining without a significant increase, with mild cytoplasmic positivity in fibroblasts and stromal cells. (b) Skin tissue demonstrating increased cytoplasmic FGF2 positivity in fibroblasts and stromal cells. (c) Thoracic tissue showing weak baseline-level FGF2 staining. (d-f) VEGF immunohistochemistry. (d) Axillary tissue demonstrating marked and intense VEGF positivity, predominantly localized in endothelial and perivascular regions. (e) Skin tissue showing moderate VEGF immunoreactivity with cytoplasmic positivity in endothelial and perivascular cells. (f) Thoracic tissue demonstrating marked VEGF positivity associated with vascular structures and stromal regions. (g-i) TGF-β1 immunohistochemistry. (g) Axillary tissue showing moderate cytoplasmic TGF-β1 positivity in fibroblasts and inflammatory cells. (h) Skin tissue demonstrating marked TGF-β1 immunoreactivity with increased staining intensity in stromal and inflammatory regions. (i) Thoracic tissue showing intense TGF-β1 positivity within fibroblast-rich stromal areas. (j-l) p53 immunohistochemistry. (j) Axillary tissue demonstrating slightly increased nuclear p53 positivity. (k) Skin tissue showing stable baseline-level p53 staining without marked increase. (l) Thoracic tissue demonstrating weak baseline-level nuclear p53 immunoreactivity.

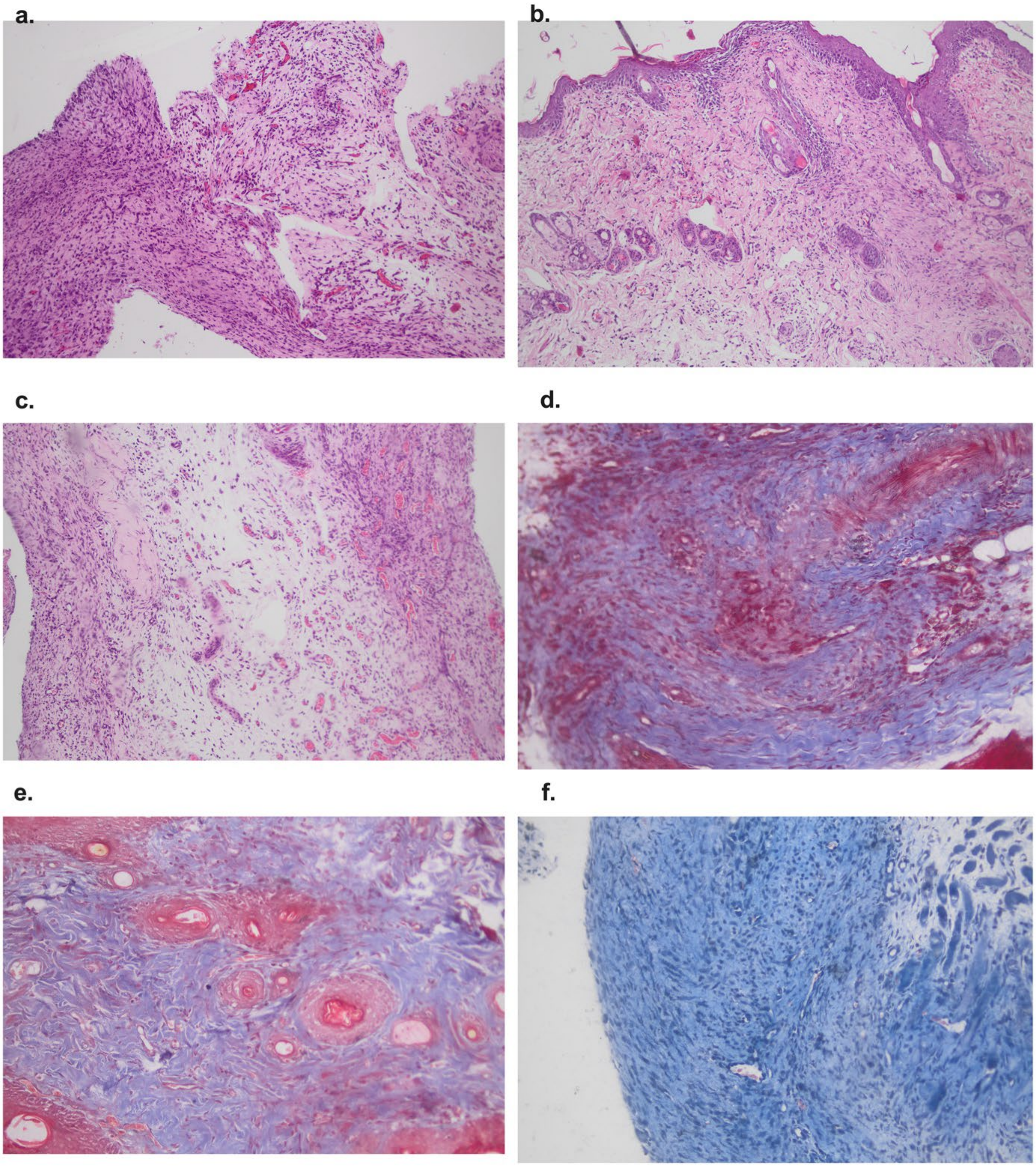

Figure S7. Representative histopathological findings in the thymoquinone (TQ)-treated group following modified radical mastectomy and axillary dissection. (a) H&E staining, TQ group, axillary tissue (×100). Prominent inflammatory cell infiltration, edema, vascular congestion/proliferation, and fibrin deposition. (b) H&E staining, TQ group, skin tissue (×100). Prominent inflammatory cell infiltration, edema, and vascular congestion/proliferation. (c) H&E staining, TQ group, thoracic tissue (×100). Prominent inflammatory cell infiltration, edema, and vascular congestion/proliferation. (d) Masson's trichrome staining, TQ group, axillary tissue (×200). Dense collagen deposition. (e) Masson's trichrome staining, TQ group, skin tissue (×200). Dense collagen deposition. (f) Masson's trichrome staining, TQ group, thoracic tissue (×200). Dense collagen deposition.

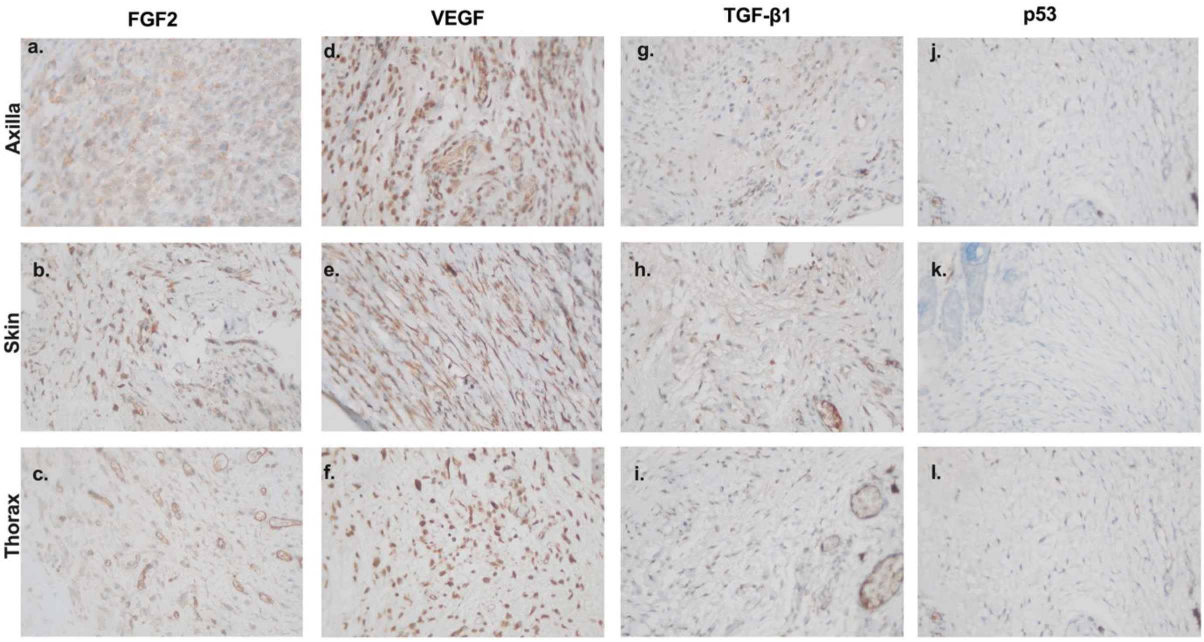

Figure S8. Representative immunohistochemical findings in the thymoquinone (TQ)-treated group following modified radical mastectomy and axillary dissection (×400). (a) FGF2 immunohistochemistry, TQ group, axillary tissue (×400). Increased positive staining, with cytoplasmic positivity in fibroblasts and stromal cells. (b) FGF2 immunohistochemistry, TQ group, skin tissue (×400). Increased positive staining, with cytoplasmic positivity in fibroblasts and stromal cells. (c) FGF2 immunohistochemistry, TQ group, thoracic tissue (×400). The most prominent positive staining observed in the study, with cytoplasmic positivity in fibroblasts and stromal cells. (d) VEGF immunohistochemistry, TQ group, axillary tissue (×400). Increased positive staining, with cytoplasmic positivity in endothelial and perivascular areas. (e) VEGF immunohistochemistry, TQ group, skin tissue (×400). The most intense positive staining (the highest expression observed in the study), with cytoplasmic positivity in endothelial and perivascular areas. (f) VEGF immunohistochemistry, TQ group, thoracic tissue (×400). The most intense positive staining (the highest expression observed in the study), with cytoplasmic positivity in endothelial and perivascular areas. (g) TGF-β1 immunohistochemistry, TQ group, axillary tissue (×400). Increased positive staining, with cytoplasmic positivity in fibroblasts and inflammatory cells. (h) TGF-β1 immunohistochemistry, TQ group, skin tissue (×400). Marked, intense positive staining, with cytoplasmic positivity in fibroblasts and inflammatory cells. (i) TGF-β1 immunohistochemistry, TQ group, thoracic tissue (×400). Marked, intense positive staining, with cytoplasmic positivity in fibroblasts and inflammatory cells. (j) p53 immunohistochemistry, TQ group, axillary tissue (×400). Slightly increased positive staining, with nuclear positivity. (k) p53 immunohistochemistry, TQ group, skin tissue (×400). Stable, baseline-level staining (no notable change), with nuclear positivity. (l) p53 immunohistochemistry, TQ group, thoracic tissue (×400). Stable, baseline-level staining (no notable change), with nuclear positivity.

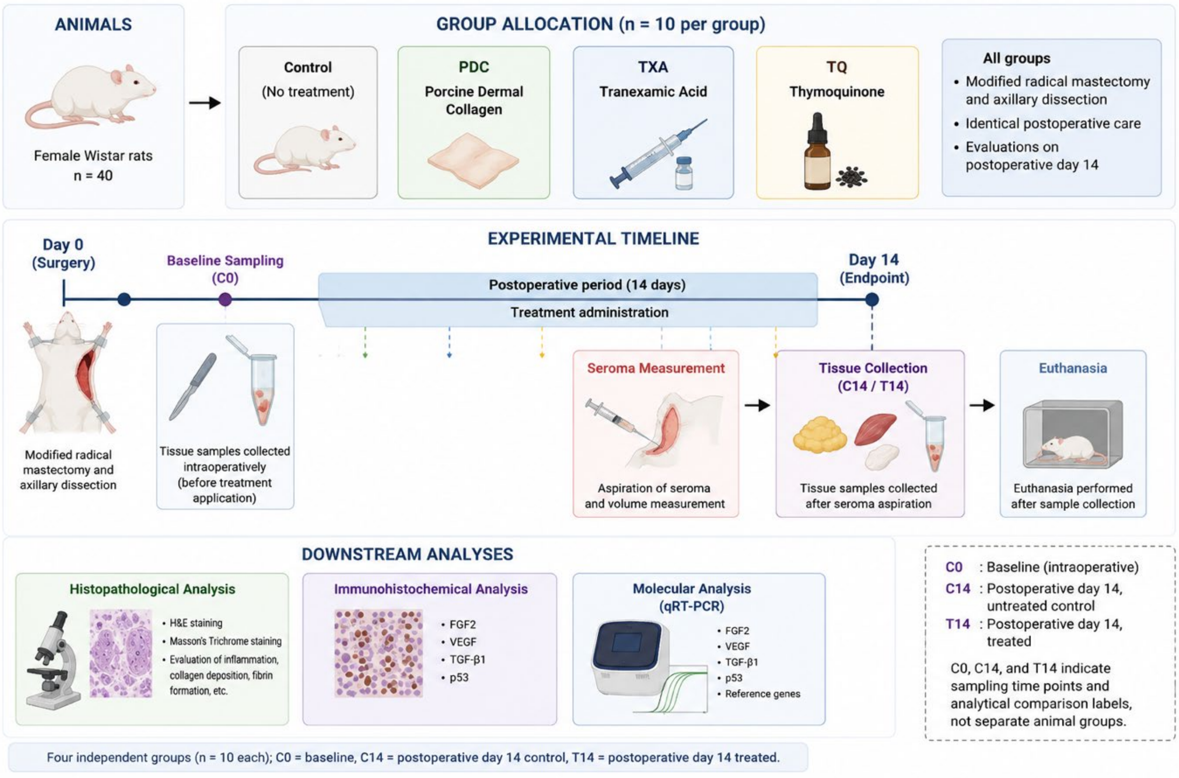

Figure S9. Workflow after experiment.

Table S1. Absolute seroma volume measurements in experimental groups on postoperative day 14.

Group	*n*	Mean ± SD (mL)	Median (IQR)	Min–Max (mL)
Control	10	5.45 ± 1.17	5.25 (4.50–6.25)	4.0–7.5
TXA	10	0.92 ± 0.18	0.90 (0.78–1.10)	0.7–1.2
TQ	10	0.47 ± 0.17	0.45 (0.38–0.60)	0.2–0.8
PDC	10	4.00 ± 1.16	4.00 (3.00–4.63)	2.5–6.5
